# Photochemical Internalization for Intracellular Drug Delivery. From Basic Mechanisms to Clinical Research

**DOI:** 10.3390/jcm9020528

**Published:** 2020-02-14

**Authors:** Waseem Jerjes, Theodossis A. Theodossiou, Henry Hirschberg, Anders Høgset, Anette Weyergang, Pål Kristian Selbo, Zaid Hamdoon, Colin Hopper, Kristian Berg

**Affiliations:** 1 UCL Medical School, London WC1E 6DE, UK; waseem_wk1@yahoo.co.uk (W.J.); c.hopper@ucl.ac.uk (C.H.); 2 North End Medical Centre, London W14 9PR, UK; 3 Department of Radiation Biology, Institute of Cancer Research, Oslo University Hospital, 0379 Oslo, Norway; Theodossis.Theodossiou@rr-research.no (T.A.T.); anette.weyergang@rr-research.no (A.W.); selbo@rr-research.no (P.K.S.); 4 Beckman Laser Institute and Medical Clinic, University of California, Irvine, CA 92617, USA; hhirschb@hs.uci.edu; 5 PCI Biotech AS, 0379 Oslo, Norway; Anders.Hogset@pcibiotech.no; 6 College of Dental Medicine, University of Sharjah, P. O. Box 27272 Sharjah, UAE; zaid19772000@yahoo.com; 7 Unit of Oral & Maxillofacial Surgery (OMFS), UCL Eastman Dental Institute, London WC1X 8LD, UK; 8 Department of Pharmacy, University of Oslo, 0316 Oslo, Norway

**Keywords:** photochemical internalization, photodynamic, drug delivery, endocytosis, lysosomes, nanotechnology, immunotoxin, nucleic acids, gene therapy, bleomycin

## Abstract

Photochemical internalisation (PCI) is a unique intervention which involves the release of endocytosed macromolecules into the cytoplasmic matrix. PCI is based on the use of photosensitizers placed in endocytic vesicles that, following light activation, lead to rupture of the endocytic vesicles and the release of the macromolecules into the cytoplasmic matrix. This technology has been shown to improve the biological activity of a number of macromolecules that do not readily penetrate the plasma membrane, including type I ribosome-inactivating proteins (RIPs), gene-encoding plasmids, adenovirus and oligonucleotides and certain chemotherapeutics, such as bleomycin. This new intervention has also been found appealing for intracellular delivery of drugs incorporated into nanocarriers and for cancer vaccination. PCI is currently being evaluated in clinical trials. Data from the first-in-human phase I clinical trial as well as an update on the development of the PCI technology towards clinical practice is presented here.

## 1. Introduction

The low cure rates for many cancer indications even after surgery, ionizing radiation and chemotherapy are, to a large extent, attributable to the limited specificity of these treatment modalities, dose-limiting toxicity, side-effects and increase of treatment resistance, all finally leading to recurrence [1,2]. This supported the development of alternative interventions with higher target tissue specificity at lower doses with fewer patient-related adverse events. These, include small molecule inhibitors of oncogenes, e.g., tyrosine kinase inhibitors, but also macromolecular therapeutics such as molecular antibodies (mAbs), oligo- and poly-nucleotides for gene modulation and nanotechnology-based delivery platforms [3]. The use of macromolecular therapeutics is restricted by the low ability of such large molecules to enter the target cells, to the extent that in the case of mAbs the therapeutic use is restricted to extracelluar targets. Weak-base drug inhibitors have also been shown to accumulate in lysosomes and therefore exhibiting reduced therapeutic outcomes but increased side effects [4,5].

### 1.1. Photodynamic Therapy

The principle of photochemical internalization (PCI) has evolved from photodynamic therapy (PDT). Thus, a brief overview of the PDT technology and the current clinical status underlining the benefits and limitations of PDT is therefore initially described. 

We summarize cancer indications where PCI may have a future potential.

PDT is a technology that has been in present in clinical research for the last 25 years. It is now commonly referred to as the fourth modality, following surgery, radiotherapy and chemotherapy, when managing a variety of tissue malignancies [6]. This intervention is based on the interaction between three factors: a drug (photosensitiser), a light (of a specific wavelength) and oxygen (in the pathological tissue). This modality can be used as a stand-alone intervention or combined with one or more of the conventional modalities without compromising them or being compromised by them. PDT is minimally invasive, unlike surgery, and can be repeated as many times as necessary with no accumulative toxicity, unlike chemotherapy. It is also a viable option where radiotherapy and chemotherapy are contraindicated, with negligible complications in comparison. Many anatomical locations have been subjected to PDT including brain, head and neck, lungs, hepatobiliary tree and the upper and lower gastrointestinal systems, male and female genital tracts, as well as pathologies of the skin, vessels, and nerves. 

PDT causes tissue destruction via the interaction between the previously highlighted three factors, which are all required for the photodynamic process. The photosensitzers used in PDT are excited by visible light to an unstable singlet state that can be converted to a more stable triplet state by inter-system crossing. The triplet state can excite molecular oxygen which is in a triplet ground state to a singlet excited state (type II reaction) and is the dominant product in PDT. Singlet oxygen oxidizes primarily cholesterol, unsaturated fatty acids, guanine and five amino acids. Alternatively, the triplet state interacts with other biomolecules by electron or proton transfer to form radicals. The basic mechanisms of PDT have been described in a large number of reviews and will not be reviewed here [6,7,8]. A large number of photosensitizers have been synthesized for use in PDT and has been extensively reviewed [9,10,11,12].

PDT includes the use of a photosensitising agent, either applied topically or administered systemically, prior to target tissue illumination with a specific wavelength of light in correlation with the absorption band of the photosensitiser. The photosensitizer is preferentially retained in the suspect tissues [13,14]. The nature of the pathological process determines the interval for maximum accumulation. Laser light can, then, be directed at the suspect tissue following a previously determined drug-light interval, thus activating the photosensitizing drug and initiating the cold (non-thermal) photochemical process. This leads to the creation of cytotoxic reactive oxygen species (i.e., primarily singlet oxygen), which induces a direct effect on tissue (i.e., necrosis and/or apoptosis) and an indirect one (i.e., impairment to tumour vascular supply and an immune response). Healing is usually fast with amazingly little scarring [15,16].

Light delivery is dependent upon the geography of the pathology. Surface illumination is generally employed for superficial and shallow disease; while interstitial light delivery is favoured towards a more profound deeper pathology, guided by endoscopic (e.g., nasendoscopy and laryngoscopy) or radiological means (i.e., ultrasound (US), magnetic resonance imaging (MRI) or computer tomography (CT)). Several types of photosensitisers are being employed with variable degrees of tumour specificity. Two generations of photosensitisers are now part of the clinical practice of photodynamic therapy in management of pre-tumourous and tumours conditions [17,18,19] and third generation PSs where the PS is linked to a targeting moiety are currently ongoing in preclinical and clinical studies (https://clinicaltrials.gov/ct2/show/study/NCT03769506) [20,21,22].

Profimer sodium (Photofrin, first production-first generation) is usually used in cases of premalignant and malignanct superficial disease involving hollow organs including gastric and oesophageal pathologies, and conditions affecting the pulmonary tree, bladder and cervix. The aim to cause enough photochemically-induced damage to eliminate the disease and prevent serious complications (i.e., structural damage to the organ wall/perforation or stenosis). This haematoporphyrin derivative has a maximum absorption at 630 nm following as a standard administration at 2 mg/kg, and a drug-light interval (DLI) of 48–72 h. The light dose was set at 50‒100 J/cm^2^, with an irradiance below 150 mW/cm^2^ [23].

As a natural heme precursor, 5-aminolevulinic acid (ALA-first generation) and the esterified 5-ALA methyl ester have been effectively used in treating skin conditions, including actinic keratosis (AK) and basal cell carcinoma (BCC), as well as oral potentially malignant disorders. With the formation of protoporphyrin IX, maximum absorption occurs at 635 nm. The drug can be administered topically in a 20% paste or systemically (60 mg/kg orally or 30 mg/kg IV). The DLI ws 3–6 h with a light dose of 100 J/cm^2^, and a rate of 100‒150 mW/cm^2^ [24,25]. Previously, it has been demonstrated experimentally that it is feasible to perform PCI with ALA-induced PpIX in cancer cells where the photosensitizer localize in endocytic vesicles [26,27]. However, this cannot be regarded as a general phenomenon as ALA-induced PpIX primarily localizes in other organelles than endosomes and lysosomes, e.g., including mitochondria [28].

mTHPC (Foscan) a potent second generation photosensitiser is regularly used in head and neck cancers to treat primary disease and in palliative care. Maximum absorption is at 652 nm with a drug dose of 0.1‒0.15 mg/kg, and a DLI of 96 h (light dose 10‒20 J/cm^2^; and irradiance of 100 mW/cm^2^) [29,30,31,32].

Second generation photosensitisers (tin ethyletiopurpurin [SnET2], mono-l-aspartylchlorin e6 [Npe6], benzoporphyrin derivative [BPD] and lutetium texaphyrin [Lu-Tex]) are currently in clinical trials, with initial results showing improved tumour specificity and shorter generalised photosensitivity. Development and introduction of newer photosensitisers and light delivery systems have substantially reduced treatment times and residual photosensitivity, while increasing the range of effective depth (i.e., necrosis and peri-lesional inflammation) [7].

Other second-generation photosensitisers include tin ethyletiopurpurin (SnET2), mono-l-aspartylchlorin e6 (Npe6), benzoporphyrin derivative (BPD) and lutetium texaphyrin [Lu-Tex] are currently in clinical trials, with initial results showing improved tumour specificity and shorter generalised photosensitivity. Further development of current photosensitisers and development of newer ones, along with upgrading the light delivery systems have substantially reduced treatment times and residual photosensitivity, while increasing the effective of response (i.e., tumour necrosis and vascular shut down) [7].

Most photosensitisers are administered systemically, usually to deal with extensive superficial disease or deep-seated pathologies. For small superficial disease, certain photosensitisers can be topically applied, and this is mainly used in dealing with dermatological conditions. The depth of response directly depends on the type of photosensitiser used and its concentration. These photochemical agents are assimilated into cellular membranes and not within the cell nuclei. The cytotoxic activity and microvascular damage achieved through the photodynamic process contribute to the direct destruction of tumour cells, and this is clinically represented as lesional/peri-lesional inflammation and swelling, subsequently followed by necrotic tissue formation. This necrotic tissue, then, sloughs away and re-epitheliasation follows as part of the normal tissue healing process. As previously highlighted, PDT is a cold non-thermal process, so no tissue heating occurs which results in the preservations of collagen, elastin and others connective tissue structures. This is an advantage of PDT when compared to interventions like conventional surgery, laser surgery, or radiotherapy where the integrity of underlying connective tissue structures is compromised [33].

Treatment periods can vary considerably depending on the absorption of light by the photosensitiser and the transfer of light energy to oxygen efficacy. In dermatology, light sources including emitting diodes and xenon are used. While coherent and monochromatic lasers are used for deep-seated disease and pathologies affecting hollow organs as can be directed and controlled when introduced along fibre-optic cables. The most commonly used lasers are diode lasers, as they are cheaper and more portable than metal vapour or tuned-dye lasers, hence became the preferred light source in photodynamic therapy. Typical treatment period for first-generation photosensitisers are about 1000 s; while second generations ones can initiate response at about 200 s of low-power laser light [7].

Previous studies have described successful use of PDT in managing primary tumours. Palliation or salvage therapy of advanced tumours to reduce bulk, restore function, control pain and bleeding, can also be achieved. It is not uncommon for the adjacent skin to appear both macroscopically and microscopically ‘normal’ and yet, it can undergo necrosis and/or apoptosis with unfavourable outcome. Such incidents are minimised by using special probes or by shielding the non-targeted tissue. Of course, ideally, a highly selective sensitizer should obviate any need to protect adjacent tissue, but at the time of writing, such photosensitizers are not yet available for routine clinical practice. Healing usually occurs with almost no scarring and the tissue architecture is also preserved as highlighted previously, which in turn supports the matrix for regeneration of normal undamaged tissue [34].

The prominent adverse events in the immediate post PDT phase include treatment-site pain and swelling. A number of studies reported that pain is commonly experienced at certain stage following PDT by nearly most patients [18,35,36,37,38]. However, most pain arises around 24–48 h following the end of light delivery and can last for up to 14 days. Pain scoring systems reported that it is usually mild to moderate in severity, which declines at the interval between necrosis and regeneration of tissues. Special pain protocols have been introduced, which mainly involved short-term non-steroidal anti-inflammatory drugs (NSAIDs) and weak oral opiates use. The pain is paralleled by a temporary rise in white blood cell levels, which is likely related to the acute inflammatory response and subsequent tissue necrosis following light delivery.

Another common adverse event when using a systemic photosensitizer is residual photosensitivity, which can last up to six weeks depending on the photosensitizer and its concentration in the skin. If the skin in not protected appropriately and the patient didn’t follow the recommended guidance for post-PDT exposure to sunlight, this can lead to skin inflammation, swelling and even superficial skin necrosis [36].

The main detriment to the PDT technology is that it requires a multi-discipline trained team and specialist equipment. Also, the photosensitisers can be quite costly. However, the benefits and advantages of these interventions are numerous, including quicker administration, less tissue damage, less adverse events, better cosmesis and quality of life when compared to the three known conventional modalities [36].

#### 1.1.1. Higher Evidence-Based Studies Required

Only one recent systematic review has been published assessing the role of photodynamic therapy in managing multiple pathologies. The review by Fayter et al. assessed the clinical effectiveness and safety of PDT in the treatment of various pathologies in various anatomical locations [39]. The outcomes measured were mortality, morbidity, quality of life, adverse events and resource use in 88 PDT clinical trials. Fayter et al. reported that PDT appeared to be superior to placebo when treating actinic keratosis; however it had better outcome when compared with cryotherapy or fluorouracil for Bowen’s disease [39]. No difference between PDT, surgery, or cryotherapy exists when it comes to response for treating basal cell carcinomas. For Barrett’s oesophagus, PDT, combined with a proton pump inhibitor (PPI), appeared to be more effective than PPI alone at long-term ablation of high-grade dysplasia and slowing/preventing progression to cancer. No firm long-term evidence could be reported for PDT in oesophageal or lung cancers. For cholangiocarcinoma, PDT was thought to improve survival when compared with stenting alone. Fayter et al. reported that there was limited evidence on PDT for brain and head and neck cancers, which was unusual due to the numerous clinical studies mainly in the head and neck field with positive outcome towards PDT [39].

The study by Fayter et al. [39] had major weaknesses such as comparing many studies based on the pathology or anatomical location disregarding the type of the photosensitizer used and its properties including the maximum absorption, dose and a drug-light interval. Also, the review did not take into consideration the light source used in each of the studies, its dose and irradiance. Suffice to say that creating a systematic review involving all pathologies treated with PDT is not statistically possible. When all relevant factors are taken into consideration, the remaining very few studies for each pathology or anatomical location will become difficult to compare. Hence, any results would actually undermine this modality and its real clinical effectiveness.

There is without doubt the need for higher evidence-based studies looking into the efficacy of PDT in comparison with other non-surgical interventions, including radiotherapy, chemotherapy and immunotherapy. A number of challenges face such studies, including funding and recruitment. Unfortunately, the only systematic review in this discipline [39] had many weaknesses, as highlighted above, and in our opinion did not give PDT a fair assessment in the field of cancer.

#### 1.1.2. The Cancer Margin

Photodynamic therapy (PDT) was designed as a minimally invasive surgical intervention in cancer management and several studies have proved its effectiveness in multi-discipline oncological care. However, more than one round of PDT maybe required due to the fact that residual tumour deposits may remain in previously treated PDT-margins leading to tumour recurrence. In a study by Jerjes et al., surgical biopsies were taken from oral squamous cell carcinoma PDT treated margins and several parameters indicating aggressive tumour growth were identified [16]. This was an issue that was never looked at before in clinical practice, but was previously highlighted in pre-clinical studies [40].

Over the last two decades, photodynamic therapy (PDT) was introduced as a minimally-invasive surgical modality in cancer management and several studies have proved its usefulness in oncological care. However, the repeatability of the treatment is usually required as residual or recurrent tumour islands have been identified in previously treated PDT-margins, where surgical biopsies have identified several parameters indicating aggressive tumour growth [16].

Norum et al. [40] has shown that PDT is less efficient in the tumor periphery (the PDT treated margin) than in the tumor center, when compared to photochemical internalization (PCI), a technology to be described next in this review. Norum et al. postulated that PCI caused larger necrotic areas and tumour regrowth in the peripheral zone was almost completely inhibited after PCI. This was followed up by a study showing that PDT had no effect on treatment of the tumor bed after surgical resection of an invasive fibrosarcoma while PCI of bleomycin caused a substantial delay in recurrence [41]. Several other studies have confirmed the improved targeting of the tumor periphery by PCI compared to that of PDT [42,43,44,45].

### 1.2. Photochemical Internalisation (PCI)

PCI or photochemical internalization is a unique intervention, which is based on the delivery of therapeutic macromolecules from endocytic vesicles and lysosomes into the cytoplasm using a sub-lethal dose of PDT. The technology has been developed for multiple purposes, but the scope of this review will only cover cancer management. PCI aims to reduce or eliminate the adverse events of most chemotherapeutics (by achieving the desirable effect with lower doses), eliminate chemotherapy resistance, reduce or avoid the skin photosensitivity, enhance the efficacy, and improve selectivity [6,46]. PCI has been tested recently in first-in-human phase one trial.

PCI reaction would usually require a photosensitive agent, which is administered systemically prior to the introduction of the chemotherapeutic agent and subsequent activation by light. The initial work on PCI suggests that this unique technology works on cellular levels in tackling pathological tissue. PCI maximizes targeted intracellular delivery of therapeutics that is not capable of breaching the cellular membranes. PCI utilizes photosensitisers that are caught in the same vesicles as the therapeutics and, following exposure to specific-wavelength light, reactive oxygen species are made, rupturing the vesicles and thereby releasing the substances into the cytoplasmic matrix, allowing the therapeutics to reach the suspect tissue.

The PCI technology is based on the controlled release of endocytosed drugs into the cell cytosol under the trigger of visible light. PCI, much like PDT, employs photosensitive drugs (photosensitisers, PSs), in this case, however, anchored within the membranes of endocytic vesicles, and which upon light activation produce ROS, predominantly singlet oxygen, shearing the membranes and causing the release of the endocytosed drugs into the cytosol. The PCI technology can make drugs normally accumulating in endocytic vesicles available to other intracellular compartments. This includes immunotoxins, genes either incorporated into engineered cationic vehicles or delivered by adeno- and adeno-associated viruses, oligo- and polynucleotides for gene modulation, as well as drugs and chemotherapeutic agents either delivered by nanoplatforms or, some of them on their own [46,47]. The intracellular delivery may be activated by external light sources, e.g., diode lasers delivering red light (600‒800 nm) that penetrate the target tissue optimally and can cover solid tumours more efficiently.

The initial mechanism and practical application was described by Berg et al. in 1999 when efficient delivery of type I ribosome inactivating proteins, horseradish peroxidase (p21ras-derived peptide) and a plasmid encoding green fluorescent protein was translocated into the cytosol in a light-dependant manner [48]. The results from this study highlighted potential clinical usefulness in cancer therapy, gene therapy and vaccination. Following this, the same group recognized the in vivo approach to site-specific cancer therapy via PCI [49]. The outcome of the study revealed a synergetic effect of combing photoactivation of photosensitizer and gelonin and the resultant PCI reaction.

Subsequently, bleomycin was presented as the chemotherapeutic agent in PCI [50]. Biochemical properties including hydrophilicity and large chemical structure makes it the ideal agent that would accumulate favourably in the endocytic vesicles with minimal leakage. The photosensitizer AlPcS_2a_ was evaluated with bleomycin to achieve the PCI effect on 3 tumours of different origins that were introduced subcutaneously in mice. The results showed delayed tumour re-growth and 60% complete response in two out of three tumour models. When compared to bleomycin alone no complete response was achieved in any tumour model.

**Figure 1:**
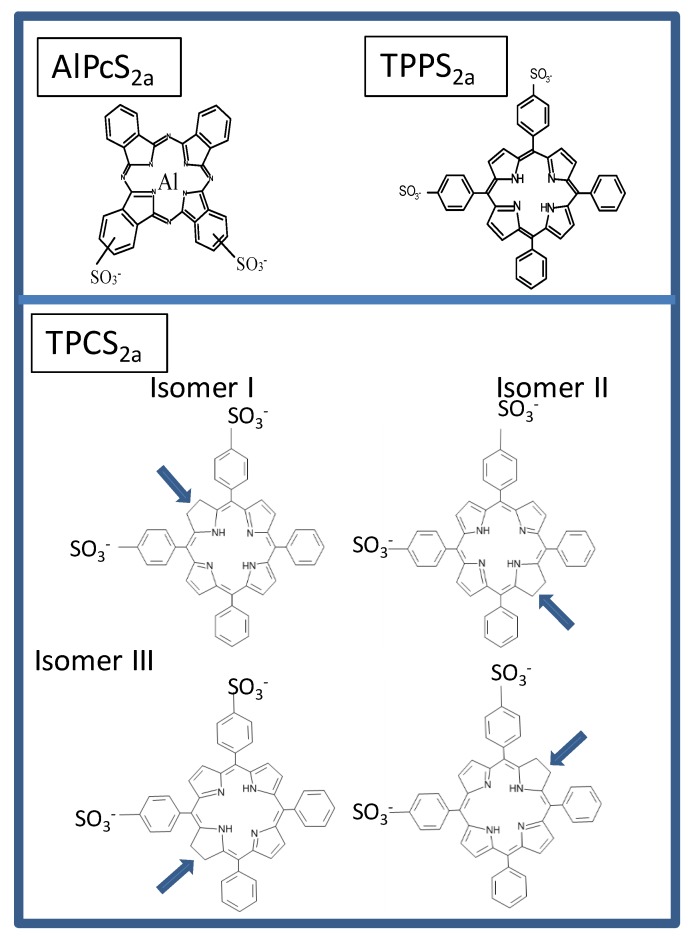
Main photosensitizers used in PCI. The reduced double bonds in the pyrrols of TPCS_2a_ are indicated. The TPCS_2a_ isomers are formed in approx. the ratio of 1:1:2 for the isomers I, II and III since isomer III can be formed by reduction of two pyrrole groups [51].

The PCI technology is currently being translated from basic research to clinical applications. A novel PS, disulfonated tetraphenyl chlorine with the sulfonate groups on adjacent phenyl rings (4, 4’-(15, 20-diphenyl-7, 8(or 12, 13 or 17, 18)-dihydro-21 H, 23 H - porphine-5, 10-diyl)bisbenzenesulfonic acid, TPCS_2a_) has been developed [51] for clinical use, and characterized with respect to its biological and photophysical/physicochemical properties [52,53,54,55,56,57]. The commercial name of the photosensitizer is now Fimaporfin while the clinical formulation is named Amphinex^®^ in which Fimaporfin is solubilized in Tween80, mannitol and Tris-buffer at pH 8.5. The structures of the main PSs relevant to PCI are presented in Figure 1, even though other PSs may also elicit PCI effects as discussed later in the text. The present review aims to provide an update on the evolution of the technology with respect to basic mechanisms, preclinical developments as well as clinical outcome. The focus of the review is on cancer treatment, even though PCI may also be applicable to the treatment of other diseases such as intracellular bacterial infections [58] and rheumatoid arthritis [59].

## 2. Basic Mechanisms

### 2.1. Cellular Uptake Mechanisms of PSs for PCI

**Figure 2:**
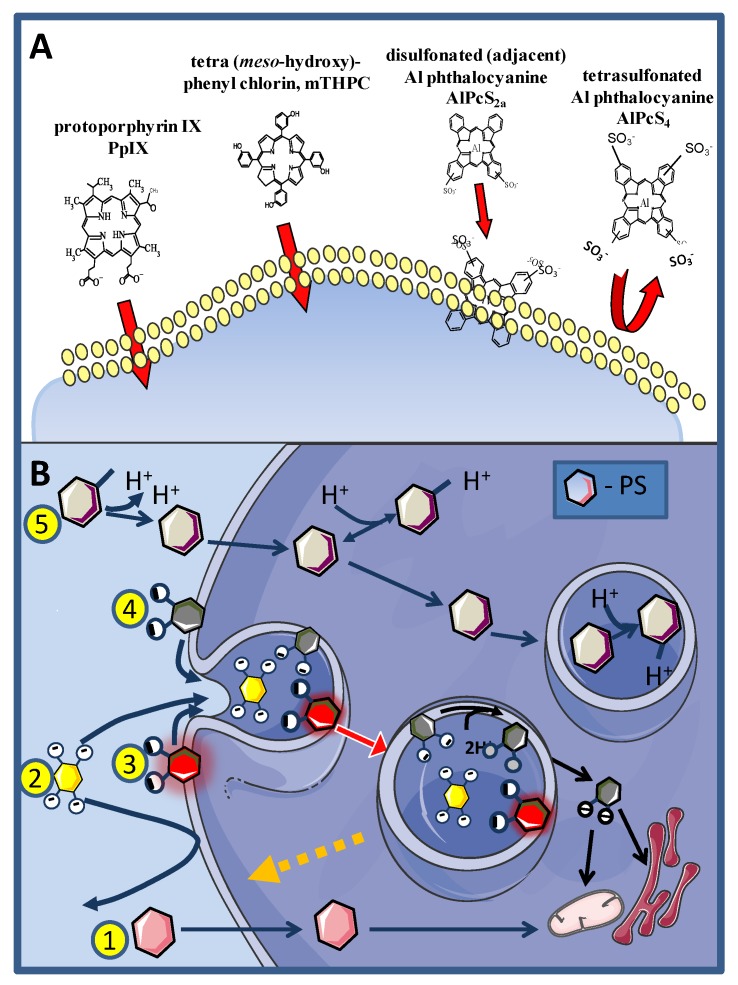
Pathways for cellular uptake of PSs. Photosensitizer penetration through a cellular membrane is influenced by its physico-chemical properties. (**A**) PSs with a low number of charged side groups, such as PpIX and mTHPC, that may be protonated at physiological pH (down to approx. pH 5 in lysosomes) are able to penetrate cellular membranes by passive diffusion while strongly amphphilic (e.g., AlPcS_2a_) and hydrophilic PSs (AlPcS_4_) will instead be taken up into cells through endocytosis. (**B**) Describes the various routes for cellular uptake of PSs. For numbering and description of the routes see the text.

Photosensitizers may enter cells either directly through the plasma membrane (Figure 2B route 1, exemplified by PpIX and mTHPC in Figure 2A) or by several endocytic pathways. Uptake through the plasma membrane may occur by simple or facilitated diffusion or by an active transport mechanism which is generally not relevant for PSs except for uptake of the PS precursor 5-aminolevulinic acid and its esterified derivatives [60].

The PSs employed to induce the PCI effect need to accumulate in endocytic vesicles and not translocate to the cytosol in the acidic conditions of late endosomes and lysosomes (typical pH range 4.0‒5.5), optimal for the function of hydrolytic enzymes [61]. Porphyrin-like PSs with only 1‒2 carboxyl groups linked to the PS core structure, for example, have a high probability of simultaneous protonation in the low pH environment of lysosomes allowing them to escape into the cytoplasm [62] (Figure 2B, route 4). PSs with more carboxyl groups or side groups with lower pKa, such as sulfonate groups, help to ensure a lasting localization in these compartments (Figure 1, routes 2 and 3, exemplified by AlPcS_4_ and AlPcS_2a_ respectively in Figure 2A). In addition, lysosomotropic weak bases (Figure 2, route 5) may also accumulate in the acidic environment of the lysosomes.

Endocytosis may occur via phagocytosis, receptor-mediated (clathrin-mediated) and non-clathrin/caveolae-mediated endocytosis, pinocytosis or adsorptive endocytosis. Pinocytosis describes the uptake of the solute surrounding the cells and highly hydrophilic PSs such as tetracarboxylic and tetrasulfonated PSs are typically taken up by cells though pinocytosis (Figure 2, route 2). The uptake efficacy, in this case, is very low and may be of the order of 100-fold lower than adsorptive endocytosis. The pinocytosed PSs are located in the matrix of the endocytic vesicles and upon light exposure run the risk of causing more damage to the contents of the endocytic vesicles (e.g., lysosomal enzymes or other endocytosed material and drugs) rather than to the vesicle membranes [63].

Adsorptive endocytosis of PSs occurs when the PSs lodge into the plasma membrane (outer leaflet in case of strongly amphiphilic PSs), without being able to transverse it (Figure 2, route 3). Typically highly amphiphilic PSs, e.g., disulfonated PSs with the sulfonate groups grouped on one side of the PSs enter the cells through adsorptive endocytosis and are, up to now, the preferred PSs for inducing the PCI effect [62,64]. Disulfonated PS with the sulfonate groups on the opposite sides of the PS do not integrate into cellular membranes and may oxidize lysosomal matrix components in a similar manner as PSs with sulfonate groups on all phenyl or phthalate ring [63].

There are two major endocytosis pathways of importance for cellular uptake of therapeutics such as in gene therapy: clathrin- and caveolae-mediated, both requiring dynamin. In both adenoviral and non-viral chitosan-based transduction/transfection PCI has been reported to enhance the gene expression only through the clathrin-mediated pathway [65,66]. These observations should not lead to the interpretation that PCI PSs do not enter caveolin-enriched vesicles which are high in cholesterol and sphingolipids (sphingomyelin and gangliosides), phosphatidylserine and phosphoinoisitides [67]. Sphingomyelins and cholesterol have been reported to suppress the disruption of endolysosomes after PDT and hence may attenuate the rupture of caveolae upon PCI [68,69].

### 2.2. Treatment Effects on Endocytic Vesicles

**Figure 3:**
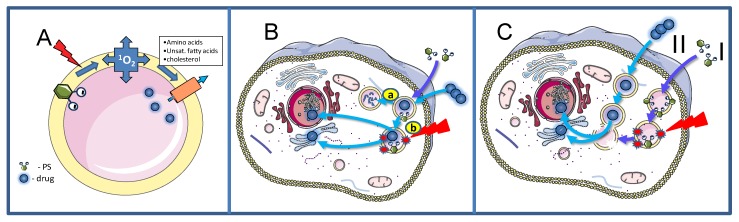
Schematic description of the PCI technology. (**A**). The figure shows an endocytic vesicle with a PS located in the vesicle membrane (inner leaflet) that upon light exposure induce ROS, primarely singlet oxygen (^1^O_2_) that oxidize biomolecules in the membrane making it permeable for the drugs in the matrix of the endocytic vesicles. (**B**). Drugs that are endocytosed may accumulate in lysosomes were they will be subjected to enzymatic hydrolyses and unable to exert any therapeutic effect (pathway a). Alternatively, drugs may be released into cytosol by means of PCI before enzymatic degradation and reach the target of their therapeutic effect in the nucleus or in the cytoplasm (pathway b). (**C**). In the “light first procedure” the PS is first administrated to the cells (step I) followed by light exposure. After the light exposure is finished the drug to be transported to the extraendolysosomal part of the cells will be administered (step II), endocytosed and fused with photochemically ruptured endolysosomes and thereby released into the cytosol according to the hypothesis.

The mechanistic background of the PCI technology is described in Figure 3. Upon exposure to light, ROS and mainly singlet oxygen are formed in the endocytic membranes where the oxygen content is higher and the lifetime of singlet oxygen longer than in the aqueous phase, strengthening the impact of the vesicular membranes in general as the main primary target of the treatment (Figure 3A) [70,71,72,73]. The singlet oxygen generated permeabilize the endocytic membranes and the contents of these vesicles including drugs entrapped in these vesicles are relased into the cytosol. From the cytosol the drugs have access to most intracellular targets into cytoplasm or nucleoplasm (Figure 3B, pathway b). In the absence of the photochemical treatrment the entrapped drugs will remain in the lysosomes or become degraded by the hydrolytic enzymes in the lysosomes (Figure 3B, pathway a). The efficacy of the photochemical treatment is enhanced by depth of penetration of the PS into the membrane [74], the membrane contents of ^1^O_2_ reactive elements such as cholesterol (may exert counter-acting effects, see below), unsaturated fatty acids with conjugated double bonds and 5 amino acids: tryptophan, tyrosine, cysteine, methionine and histidine). Lipid peroxidation chain reactions appear to be important for the rupture of the endocytic vesicles since the relocalization of the PSs to other intracellular compartments can take more than 20 min [75,76] depending on the irradiance [77], which is in accordance with the reported singlet oxygen induced lipid peroxidation chain reactions [78]. Lipid peroxidation induces changes in the membrane curvature in particular due to the intercalation of the PSs in the inner leaflet of the endocytic vesicles, that finally results in vesicle lysis [69]. Cholesterol may moderate the membrane asymmetry seen after lipid peroxidation of one of the leaflets, by a flip-flop mechanism and has been shown to reduce the membrane permeability after PDT [69]. Cholesterol is preferentially located in the plasma membrane and the early endocytic vesicles and may lead to differentiation of endocytic vesicle photochemical sensitivity [79].

Prior to the rupture of the vesicles which occurs quite abruptly [80], the intraluminal pH of the lysosomes is increasing [77]. Yao and Zhang have shown on isolated lysosomes that the lysosomal H^+^-ATPases are damaged by PDT resulting in a destabilization of the lysosomes [81] through the K^+^/H^+^ exchange [82]. The photoinactivation of the H^+^-ATPases involved cysteine cross-linking that could be reversed by dithiothreitol [83]. It should be pointed out that several other proteins were also cross-linked through formation of S-S bonds [84]. Using relatively hydrophilic dyes with low quantum yield for singlet oxygen linked to a cationic cell penetrating peptide (CPP-PS) [77] it was found that a low intralysosomal pH was necessary to induce membrane rupture and elicit release of the construct into the cytosol. This was shown by using both NH_4_Cl and bafilomycin a1 to raise the pH and is in accordance with the effect of bafilomycin on AlPcS_2a_-based PCI for transfection of cancer cells [85]. However, in contrast to the impact of NH_4_Cl on the CPP-PS mediated membrane rupture no reduction in transfection efficacy was found by NH_4_Cl on transfection efficacy by AlPcS_2a_-based PCI [85]. The reason for this discrepancy is not clear, however the interaction of the CPP-PS construct with the endocytic membranes may be influenced by the change of pH to a larger extent that with amphiphilic PSs not requiring a low pH for membrane binding [86]. The inhibitory effect of bafilomycin a1 on the AlPcS_2a_-based PCI-induced transfection efficacy was tentatively attributed to the inhibitory effects of bafilomycin a1 on endocytosis, but this has not been unequivocally confirmed. In the reports by Wang et al. [87] and Dondi et al. [88] CPP and other cationic peptides were linked to a highly hydrophobic PS that may incorporate deeper into the endolysosomal membranes and thus result in an enhanced uptake as compared to TPPS_2a_ and a strong PCI activation of the protein-toxin saporin. It has been reported that the arginine in the CPP TAT-peptide (linked to a rhodamine derivative used as PS) contributes to the destabilization of photosensitized membranes [89]. Such an effect must be indirect since arginine is most possibly not photooxidized by PDT, but more likely due to reduced CPP-binding to the cell membrane. CPP has the capacity to cross endolysosomal membranes. It has however been estimated that more than 90% of Tat-linked cargoes remain in the endolysosomal compartments [90]. Gramlich and coworkers have reported that tetanus toxin fragment C linked to the Tat-peptide along with GFP as a fluorescent marker was much more efficiently translocated to the cell cytosol following the application of PCI [91]. Accordingly, it was found that PCI of peptide nucleic acids (PNA) towards hTERT (telomerase) expression was substantially more efficient than TAT-linked hTERT PNA [92] (in the absence of PCI). Watanabe and coworkers found also that the apoptosis-inducer Bim linked to the Tat-peptide and Alexa Fluor 546 (as PS) induced a higher fraction of apoptotic cells by photochemical activation of Alexa Fluor 546 than in the absence of light [93]. Thus, PCI appears more efficient as a technology for the release of endolysosomally trapped drugs into the cell cytosol, than the TAT-peptide.

The pores generated in the vesicles following PCI treatment are large enough to release viruses such as adenoviruses (80‒100 nm) out into the cytosol, while 1‒3 m microparticles are still trapped within the PCI-compromised membranes [94].

The amphiphilic PSs used in PCI, TPCS_2a_, TPPS_2a_ and AlPcS_2a_, are strongly bound to the membranes of endocytic vesicles. Little of the activity of the matrix-resident hydrolytic enzymes found in endocytic vesicles are oxidized by the photochemical treatment although some inactivation has been reported at higher doses of light [63]. PSs that are not so strongly attached to the endolysosomal membranes can inactivate lysosomal enzymes as well as endocytosed drugs and other molecules that can be directly or indirectly oxidized by singlet oxygen. It has however been shown that performing the photochemical treatment prior to the administration of the drug of interest for intracellular delivery, may eliminate the singlet oxygen induced drug inactivation (Figure 3C). This “window” is relatively short, e.g., 5 h after the photochemical treatment the efficacy of transfection after short-pulse pEGFP/polylysine treatment is reduced by 50% [95]. The hypothesis behind this “light first” treatment is that PDT-ruptured endocytic vesicles may fuse with newly formed and intact endosomes containing cargo added to the cells after the irradiation. This would offer the undamaged contents of the newly formed vesicle, an escape route to the cytosol (Figure 3C). Foscan^®^ (mTHPC), is a highly hydrophobic and potent second-generation PS much used in head-and-neck cancer treatment (amongst others), which does not accumulate in endocytic vesicles and, hence, induces little or no PCI-like effects. Foscan^®^ has been formulated into liposomes (Foslip^®^), and PEGylated liposomes (Fospeg^®^). In contrast to Foscan^®^, both these formulations were found to induce a synergistic PCI-effect in combination with bleomycin when the light was administrated before delivery of bleomycin, but not after [96]. These results may indicate that Foslip^®^ and Fospeg^®^ enter the cells through endocytosis, inactivate bleomycin and rupture endocytic vesicles upon light exposure while bleomycin administered after the light exposure can find its way (undamaged) into the cell cytosol and reach the nucleus where it can cause DNA damage such as double-strand DNA cleavage [97]. It should be pointed out that most of Foscan^®^ in the Foslip^®^/Fospeg^®^ formulations was found to end up in the endoplasmic reticiulum as with Foscan, however, the report of Peng and coworkers [96] suggests a different route of intracellular transport for the two Foscan formulations involving endocytosis while Foscan enters the cells via passive diffusion [96].

The mechanisms of cell death due to lysosomal rupture which also apply to PCI have been presented in a large number of reviews [62,98,99] and will not be reviewed in detail here. The main cell death pathway involves the release of lysosomal cathepsins, followed by cleavage of Bid into truncated Bid (tBid) and downstream release of cyt.c from mitochondria inducing an apoptotic signaling cascade [100,101]. Cathepsins have also been reported elsewhere, to cleave the apoptosis inhibitor Bcl2, further promoting the apoptotic processes [102]. Lysosomal rupture by PDT has been shown to induce accumulation of Ca^2+^ in the cytosol, most likely released from the lysosomes, with the involvement, however, of extracellular Ca^2+^ not being excluded [87]. The increased accumulation of Ca^2+^ in the cytosol usually results in rapid sequestration of excess Ca^2+^ by mitochondria and post-PDT inhibition of this sequestration has been shown to reduce cell death, likely due to the inhibition of Ca^2+^- induced opening of the pro-apoptotic mitochondrial permeability transition pore [103].

Since all cells, except mature erythrocytes, exert endocytic activity the PSs used in PCI may also accumulate in the non-parenchymal cells of the tumor. Endothelial cells are known to exert high endocytotic activity and human umbillical vein endothelial cells (HUVECs) were found to take up TPPS_2a_ and AlPcS_2a_ at a 35-fold higher rate than HT1080 fibrosacroma cells [104]. These in vitro studies are in accordance with the high vascular shutdown observed in vivo following AlPcS_2a_- and TPCS_2a_-PDT and PCI [40,44], while the use of PCI to deliver immunotoxins to endothelial cells has shown promise [46]. Peripheral nerves are also affected by PCI as shown in a 3D co-culture system where the head and neck squameous cell carcinoma cell line PCI30 and satellite glia were more sensitive to PCI than neurons and mixed glia cells [105]. Despite the fact, however, that the neurons were less killed less by PCI, their neutrite lengths were significantly decreased after TPCS_2a_-PDT and PCI with bleomycin. These results could be linked to the high pain scores of patients treated with bleomycin-PCI, requiring the use of anesthesia during light exposure [106]

## 3. PCI Stimulated Activation of Targeted Toxins

### 3.1. Targeted Toxins

Targeted toxins are molecules consisting of one cell binding moiety and one protein toxin moiety [107,108]. The cell binding moiety, usually an antibody, antibody fragment or a ligand, recognizes an antigen preferentially over expressed on the surface of the tumor cells, while the toxin moiety, i.e., protein toxin, kills the cell. Targeted toxins emerged as a promising approach in cancer treatment already in the 1980s when whole antibodies were chemically conjugated to full length toxins. Clinical utilization was however inhibited by poor tumor penetration due to the large molecular size and the development of neutralizing antibodies. Improved molecular cloning and recombinant technology has provided a renaissance for targeted toxins. Targeted toxins can now be made smaller due to the utilization of antibody fragments, such as single chain Fv-fragments, for targeting. Decreased immunogenicity of the targeted toxins can be provided using modified toxins with immunogenic epitopes deleted. Several targeted toxins are currently under clinical evaluation and one targeted toxin, Moxetumomab Pasudotox, was approved by FDA last year for the treatment of hairy cell leukemia [109]. Within the age of personalized therapy, the development and use of targeted toxins is likely to increase significantly [108].

### 3.2. PCI Enhancement of Targeted Toxins

**Figure 4:**
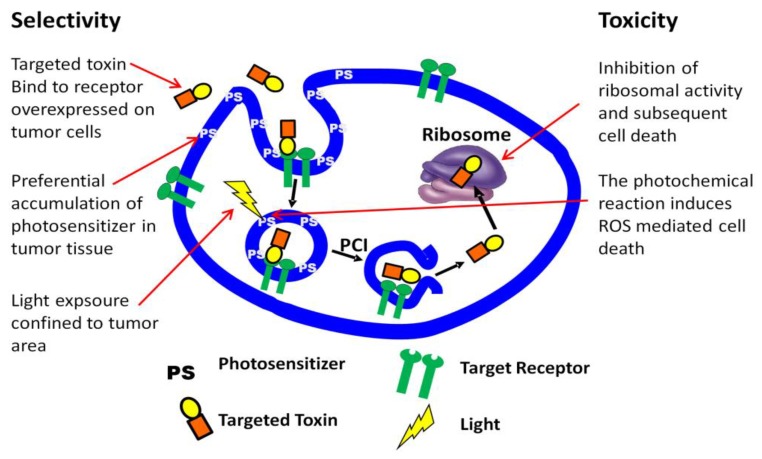
PCI of targeted toxins exerts multiple mechanisms for selective anticancer activity.

Targeted toxins, clinically approved or under clinical evaluation, are based on type II toxins intrinsically able to enter the cytosol and block protein synthesis. When these toxins are linked to a targeting moiety that is not completely specific for the target tissue substantial side effects are dose and effect limiting. Cytosolic drug delivery by PCI is theoretically most efficient when the drug to be delivered is trapped in endo/lysosome compartments without any mechanism to escape. This is fulfilled by a special type of plant derived toxins called Type I ribosome inactivating protein toxins (RIPs). These type I RIPs differ from other toxins such as ricin (type II RIP) and pseudomonas exotoxin (bacterial toxin) in that they lack a cytosolic translocation domain in addition to a cell binding moiety [110]. Type I RIPs may further be conjugated or linked to antibodies or antibody fragments recognizing antigens overexpressed in tumor tissue [111]. In combination with PCI, these targeted toxins represent a multiple of approaches to selective anticancer activity [112] (Figure 4).

The first study on PCI of targeted toxins was published already in 2000 with the type I RIP gelonin covalently conjugated to the monoclonal IgG1 antibody MOC31, directed against epithelial glycoprotein-2 [113]. MOC31-gelonin was shown to induce synergistic toxicity in combination with PCI in vitro. The efficacy was, however, limited by low rate of endocytic uptake of the immunoconjugate through its surface receptor and the project was not continued in vivo [112]. Effective endocytosis of the targeting toxin is a prerequisite for PCI enhancement and several different immunoconjugates have been tested. The strong non-covalent biotin/streptavidin linkage has been widely used to link different antibodies to the type I RIP saporin in order to generate targeted toxins for PCI evaluations [114,115,116,117,118,119]. In these studies, epidermal growth factor receptor (EGFR), HER2, and the cancer stem cell marker CD133 stood out as promising targets for further development of PCI enhanced targeted toxins.

Although highly convenient for in vitro screening of targeted toxins for PCI delivery, the streptavidin-biotin linkage is not suitable for in vivo applications, among others, due to the large size of the generated immunoconjugates. The optimal size for efficient delivery of protein based macromolecules to tumors has been estimated to 50‒100 kDa, smaller proteins will be subjected to renal excretion [120,121] while larger proteins will have lowdiffusion rates in tumors and limited penetration efficacy [122,123]. Type I RIPs are approximately 30 kDa [110] while a full antibody is 150 kDa. The optimal size of targeted toxins may be reached by genetic fusion of the Type I RIP and small sized targeting moiety of interest, e.g., a scFv fragment of an antibody or an endogenous ligand. The first recombinant targeted toxin used in vivo in combination with PCI was scFvMEL/rGel targeting CSPG4 [124]. Complete regression was observed in 33% of the nude mice bearing amelanotic melanoma (A-375) xenotrafts after only one treatment cycle, while only minor tumor growth delay was observed with scFvMEL/rGel or photosensitizer and light (photodynamic therapy) as monotherapies.

### 3.3. PCI of Recombinant Targeting Protein Toxins; Current Status

During the last years PCI of targeted toxins has been demonstrated as an efficient modality in several cancer models. We have been focusing on three recombinant targeted toxins; MH3B1/rGel targeting HER2 [125,126], vascular endothelial growth factor fused to recombinant gelonin (VEGF121/rGel) [42,44] and epidermal growth factor fused to gelonin (EGF/rGel) [43].

HER2 is overexpressed in 20%‒30% of breast cancer and is associated with aggressiveness [127,128]. Our first in vitro data on MH3B1/rGel-PCI was highly promising indicating this treatment approach as highly efficient in both HER2 high- and low-expressing tumors [125]. MH3B1/rGel-PCI was later evaluated in a HER2 expressing model in vivo, but was found less efficient than other targeted toxins tested [126]. Studies on MH3B1/rGel-PCI have therefore not been continued after this.

EGFR is over expressed on the surface of cancer cells from several origins and is one of the most studied surface receptors for targeted cancer therapeutics. Despite the quality of our first version of EGF/rGel which suffered from impurities and significantly reduced gelonin activity as compared to unmodified gelonin, EGF/rGel-PCI was shown highly tumor specific and potent in two xenograft models [43]. EGF/rGel is currently redesigned to optimize this targeted toxin for PCI mediated delivery.

VEGF121/rGel was first produced by Dr Rosenblums laboratory at MD Anderson Houston Texas as a vascular targeted toxin [129]. VEGF121/rGel as monotherapy exerts its action mainly through VEGFR2 which is over expressed on tumor endothelium [130] and it was expected that PCI could increase this vascular response. VEGF121/rGel-PCI was indeed shown to exert high efficacy on VEGFR2 expressing endothelial cells and was also shown to increase the vascular damage of CT26.CL25 xenografts in mice [44]. Our recent data have, however, revealed that VEGF121/rGel also induce a direct cytotoxic effect on the tumor parenchymal cells and that complete remission, which is observed in ~50% of the animals, is highly dependent on a T-cell mediated immunoresponse [42]. We are currently further developing VEGF121/rGel in preclinical models.

The cancer stem cell (CSC) hypothesis is based on the assumption of treatment resistant and highly aggressive tumor initiating cells, which need to be killed for tumor eradication. We have in several projects evaluated PCI of CSC-targeting therapeutics, and our results clearly demonstrate that PCI of targeted toxins can circumvent CSC resistance [130]. We are currently designing a recombinant CSC targeted toxin for PCI-mediated delivery, which is a prerequisite for further development of this concept in preclinical models.

## 4. Nanoparticle-Mediated PCI as a Powerful Anticancer Drug Delivery System

As PCI is a technology based on endocytosis, it has been more applicable to therapeutic macromolecules like bleomycin [50,97,106] or gelonin [46]. Most of the mainstream, efficacious anticancer therapeutics doxorubicin, etoposide, tamoxifen, irinotecan, etc. are, nonetheless, relatively small molecules with a molecular weight around or less than 500 Dalton and also exert other characteristics of compounds that easily penetrate cellular membranes [97,131,132]. Since the inception of PCI as a targeted anticancer drug delivery strategy, the above incompatibility created a need to find delivery platforms which will render PCI applicable to small-molecule therapeutics.

There are numerous drug delivery systems based on nanotechnology which produce great results on the bench, yet fail to advance to the clinic. The main reason for this is their lack of specificity and targeting to cancer tissues. Most of these formulations spontaneously release their cargo at a time-dependent manner when they are internalized by cells, whereas in the clinic, a trigger is required so that only the nanoparticles taken up by tumour cells can release their load. Such a system would require a tight sequestration of the drugs within the nanocarriers until an external trigger elicits the cargo release.

PCI fulfills the criteria of the above described external trigger by spatial selection, with a clinically needed simplicity: Light illumination confined in the tumour area (including also a normal tissue margin) can guarantee that only the endolysosomes in the illuminated region will be ruptured and the trojan-horse nanoparticles will only release their cargo within the cells resident in the irradiated region. Recently, there have been various attempts in the literature to develop nanoparticles custom-made for PCI-mediated drug delivery.

In a study by Sauer et al. [133] colloidal mesoporous silica nanoparticles (NPs) had their thiol-functionalized cores reacted with cysteine forming disulfide bonds. The NPs were endocytosed by HuH-7 human hepatocellular carcinoma cells and upon Amphinex^®^ (TPCS_2a_) -mediated PCI, the NPs were released into the cell cytosol where intracellular thiols and primarily glutathione (GSH) reduced the disulfide bond, releasing the cysteine cargo. Although this study did not directly address the activity of any anticancer agents, it illustrated the potential of the nanoplatform employed as an efficient drug carrier for PCI since the endolysosomal compartments express low reductive capacity leaving the disulfide bridges uncleaved. The disulfide strategy in two other studies one with camptothecin linked to thiolated PEG-P(lys) copolymers [134] released into cytosol by photoactivation of Photofrin and the other releasing doxorubicin (DOX, a potent DNA intercalator and topoisomerase II inhibitor) from arginine terminated poly (ester amide) backbone linked to hyaluronic acide-AlPcS_2_ via a disulfide bridge [135]. In both cases synergistic effects were observed.

In another study employing redox-sensitive NPs, Zhu and coworkers, developed *N,N*-dimethylacrylamide (DMA) polymers based on eosin as the photosensitizer covalently conjugated, while CPT was also linked to the backbone by disulfide bonds [136] to be cleaved of with intracellular GSH. The addition of rare-earth-doped upconversion nanoparticles yielded hybrid vesicles. Indeed, PCI at 980 nm downconverted to excite eosin, employing the polyDMA NPs, conferred a profound photochemical toxicity to HepG2 human liver carcinoma cells, which increased with increasing 980 nm irradiation dose and CPT concentration.

Lu et al. [137] implemented polyion complex (PIC) micelles through electrostatic interactions between dendrimeric phthalocyanine (DPc) and poly (ethylene glycol)-b-poly(l-lysine) block copolymer (PEG-PLL). These micelles were used for the delivery of DOX, via PCI on DOX-resistant MCF7 cells, both in vitro and on mouse xenografts. PCI produced better results when phthalocyanine light activation took place before DOX administration (“light first”), both in vitro and in vivo. Basic drugs, like DOX, mitoxantrone, etc., are protonated upon entry in acidic cell endocytic vesicles, therefore become unable to cross the membranes and consequently staying sequestered therein [138,139,140]. The study by Lu et al. [137] used the phthalocyanine-based nano-construct mostly to rupture the cell endosomes, rather than to smuggle DOX into them for PCI purposes, as “light first” treatment worked better: Compromised endocytic vesicles could no longer retain DOX, which found its way to the nucleus. This is in accordance with a previous study in DOX resistant cells due to exceptionally low lysosomal pH where DOX was relocated to the nucleus after photochemical damage of the lysosomes [141]. In a similar study, the use of a complex of PIC and homocatiomers (a catiomer (cationic polymer) derived from the repetition of only one species of cationionic monomer) loaded with AlPcS_2a_ led to a three-fold enhancement of the AlPcS_2a_ uptake and also of the photosensitivity, compared to free AlPcS_2a_ in human alveolar lung adenocarcinoma A549 cells [142].

DOX was also used as the anticancer agent of choice in polymers based on chlorin-e6 as the photosensitizer (PS) and pluronic F-127 as the amphiphilic copolymer, self-assembling intoDOX-loaded micelles [143]. These micelles exhibited enhanced singlet oxygen generation as compared to free chlorin e6 in aqueous media, and a clear PCI effect was noted in HCT-8 human colon adenocarcinoma cells increasing with the irradiation dose for DOX-loaded micelles, but not with free DOX in vitro and also in a UV-2237 drug-resistant murine fibrosarcoma model in nude mice. The in vivo study would be more informative if a free DOX + free chlorin e6 PCI group had been included.

Pasparakis et al. [144] developed NPs based on an acetal polymer exhibiting a low photolysis threshold as well as a tendency to acidolysis in acidic environments (e.g., lysosomes). The polymer was also modified by introducing a 2-nitroresorcinol comonomer to “red-shift”/spectrally extend its photolysis from the UV (365 nm) to the green (532 nm) compatible with the second harmonic of a Nd:YAG laser. CPT and hematoporphyrin (HP, the PS) were chosen for encapsulation in the polymers. PCI application by irradiation significantly reduced HeLa cell viability in comparison to free CPT or the combination of HP/CPT without light. In our opinion, the addition of an acidolytic polymer functionality could have been avoided since it may have facilitated a spontaneous CPT release within endocytic vesicles indiscriminately. The photolytic property of the NPs, on the other hand, had it been matched to that of a PS further spectrally shifted to the red (>650 nm), and hence more clinically relevant, would have been perfect to unleash the drug in a “double unwrapping” action: from the endosomes and the NPs at the same time.

In the study by Liu et al. [145], the authors have developed NPs from double hydrophilic block copolymers bearing the eosin Y photosensitising moiety within the pH-responsive block. These NPs showed a capacity to modulate singlet oxygen with decreasing pH upon the micelle-to-unimer transition below ~pH 6. Once again, the drug of choice was CPT and a pronounced PCI effect was shown on A549 alveolar lung adenocarcinoma cells for NPs loading with CPT, following irradiation in the green spectral region (520 nm). The results of this study would have been substantially reinforced by inclusion of the effect of free CPT + free eosin application on the A549 cells.

Theranostic approaches have attracted much attention. In the report by Liu et al. [146] fluorescent graphitic hollow carbon nitride nanospheres (GHCNS) were implemented to simultaneously serve as PCI photosensitizers, an imaging agents and a drug carrier. The GHCNS were externally functionalised with hyaluronic acid (HA) to develop an affinity towards cells with overexpressed CD44 glycoprotein, like MDA-MB-231 human breast ductal adenocarcinoma cells. The strategy was for HA to be digested by hyaluronidase (hyal) available in endocytic vesicles [147], and consequently the GHCNS-HA to be “uncapped” and their cargo released. DOX loaded GHCNS-HA were reported by the authors to elicit a PCI effect in vitro which was, however, not substantially higher than the dark effect of the NPs. It is quite possible that the formulation could very much benefit from an externally administered PS and/or a different capping strategy of the nanospheres.

Another approach to nanocarrier-assisted PCI was presented by Yaghini et al. [148], where the authors used liposomes surface-functionalised with a cell penetrating peptide (CPP). The CPP had additionally a porphyrin moiety (meso-tetrakis tetraphenylporphyrin (TPP)-derivative) as the PS, conjugated to its N-terminus. The liposomes were loaded with saporin, a ribosome inactivating protein. Saporin PCI conferred enhanced cytotoxicity to MC28 cell cultures increasing with saporin concentration and irradiation dose. The study would have benefited from a free TPP + saporin PCI control to further show the advantage of using the liposomes as saporin carriers. Liposomal carriers may exhibit variable responses with PCI depending on their surface charge and functionalisation but also on their lipid content. For example, liposomes with cationic charges on their surface are more likely to undergo adsorptive endocytosis enhancing cellular uptake, however this also depends on the distance of the charge from the surface [149]. Martínez-Jothar and coworkers [150] synthesized PEGylated poly(lacticacid-co-glycolic acid-co-hydroxymethyl glycolic acid)(PLGHMGA) NPs, surface-functionalised with the 11A4 nanobody that is specific for the tyrosine-protein kinase erbB-2 (HER2) receptor for the targeted delivery of saporin to HER2 positive cancer cells. The combined use of saporin-loaded 11A4-NPs and PCI strongly inhibited cell proliferation on HER2-positive cells only and decreased cell viability through induction of apoptosis, showing a substantial improvement from PCI on free saporin.

Cheng et al. [151], tried a different approach where they functionalised murine exosomes with a “ChiP” consisting of chimeric peptide for targeting both the plasma membrane and the nucleus (by nuclear localisation sequence (NLS)), and protoporphyrin IX (PpIX) as PS. This functionalisation aimed at helping the exosomes anchor onto the cell membranes and enter the cells through endocytosis, to be released into the cytosol by PCI and enter the nucleus by means of the NLS sequence. A second irradiation leads to the disruption of the nucleus due to the photogenerated ROS formation. This study demonstrates that exosomes have the potential of becoming PCI-Trojan-horses, especially for transporting chemotherapeutics to the cell nuclei, e.g., DNA intercalators and topoisomerase I and II inhibitors.

Luo et al. [152] implemented a nanosystem comprising of a photosensitizer, TPPS_2a_, and catalase in the inner core, DOX in the polylactic polymeric shell via a ROS cleavable thioketal bond (TK) and the IF7 peptide on the surface which specifically binds to annexin I. While free DOX did not elicit any notable cytotoxicity in the absence of irradiation in DOX-resistant MCF7/ADR cells, DOX loaded NPs showed a profound PCI cytotoxicity (~90% at 3 ng/mL DOX). In human umbilical vein endothelial cells (HUVEC), the NPs photochemical toxicity was similar, but in this case free DOX also induced a profound toxicity on the cells (~60% at 3 ng/mL DOX) as expected. This was also reflected in vivo, through a PCI induced profound tumour growth inhibition onmice MCF7/ADR xenografts resulting in a considerable survival benefit. This system represents a successful example of a “double photo-unwrapping” PCI trojan horse.

In the work by Tian et al. [153] a pH-sensitive amphiphilic co-polymer block linked via an acetal group to 5-(4-Hydroxyphenyl)-10, 15, 20-triphenyl-porphin (TPP-OH) as the photosensitizer, was used to self-assemble into micelles which were further loaded with DOX. Despite the fact that no notable PDT effect was observed for up to 21.1 μg/mL (~34 μM) of porphyrin content at 420 nm, there was a slight PCI effect for DOX concentrations > 1.56 μg/mL (TPP-OH > 0.66 μg/mL) especially in the case of the acetal bond with the TPP where the effect was more pronounced. The low level of PDT effect of TPP at 34 μM suggests a tight sequesteration of the polymers in the endocytic vesicle matrix. The acetal bond could be acidolysed therein to release some of the TPP which then gets incorporated into the inner endocytosome membrane to slightly increase both the PCI and PDT effects (±DOX), in comparison to the non-acetal polymer.

Jin et al. [154] employed low density lipoprotein (LDL) loaded with fluorescent dyes in three different ways: (i) protein labelling of LDL (LDL-FITC) by conjugating fluorescein to the lysines of the LDL apoB protein, (ii) surface loading of LDL NPs with the lipophilic Dil stain (Dil-LDL) and (iii) LDL NPs core loading with hydrophobic fluorescein conjugated to two oleyol groups (LDL-Fluo-BOA). PCI application on the NP loaded cells with AlPcS_2a_ on A549 alveolar lung adenocarcinoma cells resulted in efficient cytosolic release, predominantly of the surface loaded but also of the protein loaded cargo, but no cytosolic escape of the hydrophobic dye was noted following PCI in the case of the core-loaded NPs.

Theodossiou et al. [155] developed a bimodal molecule through the covalent conjugation of a 5, 10, 15, 20-Tetrakis-(3-hydroxyphenyl)-21, 23H-porphine (mTHPP) moiety to a β-cyclodextrin (mTHPP-βCD). Upon complexation with tamoxifen (TAM), a selective estrogen receptor modulator, a strong complex was formed which was not dissociated in the presence of serum. Application of mTHPP-βCD-TAM based PCI conferred high photochemotoxicity to estrogen-receptor-positive MCF7 cells which was not abrogated 72 h post irradiation. A smaller scale effect was also noted in the triple negative MDA-MB-231 cells, however the cells recovered for the most part within 72 h from the irradiation. Moreover, mTHPP-βCD-TAM PCI collapsed cellular respiration of MCF7 cells to less than half the basal rate, merely 1 h following cell irradiation. The critical issue with such a complex s mTHPP-βCD is whether the CDs can efficiently retain their guest cargo in the clinical setting, i.e., in the blood circulation, and yet release it readily upon the PCI trigger. In the present case [155] it seems that the PCI-mediated disruption of the endocytic membrane integrity and abrupt release of endolysosomal contents, shifted the equilibrium to favour dissociation of the complexated TAM from its βCD host. The fact that unlike TAM-FITC fluorescence which after irradiation relocated to cell mitochondria the mTHPP fluorescence remained punctate, suggests a tight anchoring of the mTHPP-βCD conjugate even when the endocytic membranes are ruptured, indicative of a system for pure PCI drug delivery, without much of a secondary PDT effect due to PS relocation.

Another approach was to utilize PCI for diagnosis in vivo [156]. Kim et al. prepared two types of nanoparticles consisting of Gadolimium (Gd) chelates within either hydrophilic (pullulan-DTPA-Gd, PDG) or amphiphilic (pullulan-DOCA-DTPA-Gd, PDDG) polymers. The idea was to create new contrast agents, which indeed exhibited higher contrast intensity than the Gd-DTPA chelate. PCI using pheophorbide-a as the photosensitiser, largely enhanced the intracellular uptake of PDG and PDDG with PDDG (amphiphilic) exhibiting ~3-fold efficiency over the hydrophilic PDG. The labelling efficacy of the two compounds in hMSCs with PCI were also tested in CT26 murine tumour models, by injection of the Gd-polymer-loaded hMSCs, showing a low exocytosis and increased, long-lasting contrast in the tumours.

From all the above studies reviewed, it is evident that the most preferred anticancer agents were DOX, CPT and the ribosome inactivating protein (RIP) saporin, while only a couple of studies involved different drugs mainly TAM and 5-FU as well as Gd-NPs as an interesting diagnostic application for MRI imaging. There were also some prominent and recurrent secondary strategies employed to assist PCI, namely (i) pH-responsive and (ii) intracellular GSH reducible disulfide bond architectures. Regarding the acidolytic bonds, it has to be said that the main danger is for the drugs to escape the endocytic vesicles of both cancer and normal cells indiscriminately prior to the application of PCI, except if the drugs are chosen to be such that will not escape the lysosomes (e.g., saporin that will be fully trapped within lysosomes). In addition, polycationic or polyamine NPs should be used with caution as they could trigger the proton sponge effect [157] causing the endocytic vesicles to undergo spontaneous endosomolysis, becoming leaky and consequently releasing their sequestered cargos without any external control.

The disulfide bridge strategy is also a very interesting approach to increase PCI efficacy, however the main danger is the spontaneous reduction of the S-S before the NPs reach the endosomes. This could for example take place in the blood circulation, where although the ratio of reduced to oxidized thiols is low, there are still reduced thiols present, with serum albumin thiol (HSA-SH) being the most abundant [158].

Another interesting strategy used for better penetration of light within the tissue is the use of up-conversion nanoparticles, which can significantly increase the reach of light into tissue e.g., in the case of external irradiation at 980 nm. This also raises another issue with the construction of nanocarrier strategies for PCI as reviewed herein. Several studies use PSs which are not clinically relevant as they absorb at very short wavelengths with even submillimeter light penetration into tissue. For reasons of clinical applicability, it is important to focus on photosensitisers with long activation wavelengths and high singlet oxygen quantum yields. Even porphyrins have a handicap in this respect in comparison to their chlorin counterparts as the latter usually exhibit up to ~10× higher red absorption Q-bands.

In our opinion, the best strategy for the use of nanocarriers in the anticancer arena, is the double unwrapping strategy where light of the same wavelength ruptures the endosomes and also cleaves photolabile bonds of the NPs simultaneously. This can help avoid the bottleneck of endocytic trapping of the NPs/drugs but also the spontaneous leakage of the drugs from endolysosomes unrelated to the PCI stimulus

Another exciting PCI application in nanotechnology is the use of PCI-inducible nanoplatforms to enhance gene delivery into the cells. This could mean improvement of (i) DNA delivery to transfect cells and induce the expression of a new protein, (ii) small interfering RNA (SiRNA), oligonucleotides, morpholinos (chemically synthesized targeted oligos), and (iii) mRNA in order to modulate the expression of a protein in the cells, or silence a gene of interest.

## 5. PCI Delivery of Oligo- and Polynucleotides

Being large and generally charged molecules, nucleic acids are not able to pass through the plasma membrane of cells. Although fusogenic delivery vehicles or technologies inducing physical permeabilisation of the cell membrane have found a certain use, most nucleic acid delivery systems at some stage involve endocytic uptake, either of vehicle/nucleic acid complexes, of viral vectors, or in some cases, of naked nucleic acid molecules. For most such systems endosomal escape of the nucleic acid constitutes a very important delivery barrier, thus, as an endosomal escape technology, PCI has a large and broad potential in the area of nucleic acid delivery. In accordance with this strong enhancement of nucleic acid delivery by PCI has been demonstrated across a broad spectrum of nucleic acids and delivery vehicles, ranging from naked oligonucleotides, through mRNA and plasmids to different types of viral vectors. Along the lines of using PCI to more efficiently introduce foreign genetic material into cells to modulate or alter their gene expression, numerous nanocarriers have been employed to transport siRNA, mRNA and-or oligonucleotides, in order to deliver new genetic material or to silence targeted native genes.

In the following paragraphs, the use of PCI for the delivery of various types of nucleic acids will be discussed.

### 5.1. PCI Mediated Oligonucleotide Delivery

Several types of oligonucleotides have a great therapeutic potential for the use in down-regulating gene expression or for modifying RNA splicing, and several therapeutic oligonucleotides are already on the market. Delivery of oligonucleotides to other organs than the liver is however still a major problem. PCI-induced release of an oligonucleotide from endosomes was demonstrated for the first time by Høgset et al. [159]. Since then several groups have shown that such release also leads to enhanced biological activity of many different types of oligonucleotides. Folini and co-workers [92] used PCI with a naked peptide nucleic acid (PNA) to show that the PCI technology enhanced the biological activity of a PNA directed to the hTERT subunit of telomerase. Shiraishi and Nielsen [160] and Bøe and Hovig [161] further showed that PNAs conjugated to cationic peptides for the enhancement of cellular uptake were excellent substrates for PCI, with an PCI-induced enhancement of biological activity of > 100 times being achieved with some of the PNA constructs [160], also in accordance with other reports [162].

In an interesting study Bøe and co-workers [163] demonstrated light-induced endosomal release and gene silencing for a PNA oligonucleotide where a photosensitizing molecule was covalently attached to the oligonucleotide. The construct also included a positively charged nuclear localization signal peptide, thus constituting a multi-functional molecule offering cellular uptake, gene silencing, and the possibility for light-induced endosomal escape in one cargo.

Further studies on using PCI for oligonucleotide mediated gene expression silencing have been performed with siRNA in combination with a variety of delivery vehicles. Thus, Bøe and co-workers showed good effects of PCI with siRNA with polymeric vehicles such as polyethylenimine [164], cyclodextrin-containing polymers [165], peptides [166] and polyamidoamine (PAMAM) carriers [167]; as well as with some, but not all of the tested lipid vehicles [164]. In case of PAMAM dendrimer complexed the authors tested several generations (0‒7) of PAMAM dendrimers complexated with both a GFP siRNA and a GFP mRNA with regard to their PCI-mediated gene modulation efficiency in OHS-EGFP and OHS osteosarcoma cells respectively. The PCI induced downregulation of GFP expression in the stably transfected OHS-EGFP cells was remarkable for PAMAM generations G3 and above (80%‒90%) and especially since the cytotoxicity could be less than 10%. Interestingly GFP upregulation by delivery of EGFP mRNA, was not achieved with any of the PAMAM dendrimeric carriers investigated, neither with nor without the application of PCI. PAMAM dendrimers have also been successfully shown to carry splice switching oligonucleotides activated by PCI as seen by nuclear splice swithing activity [168]. For peptide vehicles, most of the poly(l-arginines) tested and some poly(l-lysines) showed a considerable PCI effect in s100A4 silencing in OHS cells, while poly(l-histidine) carriers were not so effective [167]. In contrast to the findings by Bøe et al. [164], Oliveira and co-workers [169] also demonstrated good effects of PCI for lipofectamine mediated siRNA delivery, and most notably were able to show that this combination also could give strong downregulation of EGF receptor expression in vivo, leading to tumour growth retardation in an EGF dependent tumour model [170].

Using nanogels based on dextran and methacrylate derivatives for siRNA delivery and as an siRNA depot inside endosomes, Raemdonck et al. [171] showed that it was possible to prolong the knockdown of the target protein in a cell by using PCI for releasing siRNA from endosomes up to a week after transfection.

Varkouhi and coworkers [172] used two biodegradable cationic polymers, one based on methaclylamide (pHPMA-MppM) and the other based on chitosan (TMC), to complexate with luciferase siRNA. The silencing activity and the cytotoxicity of these polyplexes were compared to polyplexes of non-biodegradable methacrylate (pDMAEMA) and polyethylenimine (PEI) polymers. The PCI effect on the gene silencing was compared to that of an endosomolytic peptide (dilNF-7), and both were found to increase the silencing of all the polyplexes to 70%–80% except for PEI where PCI did not enhance the treatment effect. It should be pointed out that PCI of PEI polyplexes are most efficient at lower N/P ratio than usually used (see also below).

Zhang et al. [173] developed upconversion core-shell NPs which they named orthogonal photoactivatable superballs (OP-SBs). The OP-SBs were based on two different upconversion NP moieties mixed in different ratios: one resulting in high absorption at 980 nm (high tissue penetration) and strong red-light emission and another one, absorbing at 808 nm and emitting in the blue and UV. The therapeutic strategy was a dual one combining the singlet oxygen production from the OP-SBs coated with a thin mesoporous silica layer, functionalised with azobenzene and then loaded with Zn-phthalocyanine, and a PCI-induced knockdown of SOD in the cells using siRNA. While PCI induced a sizeable knockdown on the SOD levels in all cell lines tested the viability was not profoundly different between the PDT and PCI groups in vitro. The PCI effect was better when both the 980 and 808 nm excitations were used. In nude mice, human oral adenosquamous carcinoma cell (Cal-27) xenografts PCI showed superior tumour suppression efficacy again more pronounced with sequential 980 and 808 nm excitation.

Core-shell upconversion nanoparticles were developed by one-pot synthesis by Jayakumar et al. [174]. These nanocarriers could upconvert deep tissue penetrating 980 nm light into blue light able to excite TPPS_2a_ (413 nm) for PCI, but also into UV light which could cleave a photolabile tether releasing a STAT3 antisense morpholino to knockdown STAT3. The strategy was tested in B16F0 mouse skin melanoma, where it was found that light activation increased STAT3 knock down both in the absence and presence (PCI) of TPPS_2a_, however the PCI-effect was significantly higher. In vivo, on a mouse melanoma model, a bigger STAT3 knockdown was registered for the PCI group. In addition, there was a significant tumour growth inhibition in the NP-treated and again the PCI group exhibited the biggest tumour suppression.

In the work by Ekiner et al. [175] mesoporous organo-silica nanoparticles (PHT-PMO) were prepared based on a silylated Zn-phthalocyanine precursor. PHT-PMO NPs elicited no dark toxicity to MCF7 human breast adenocarcinoma cells but were highly phototoxic atwhen irradiated at 650 (71% death) and 810 nm (63% death) and even more so at 760 nm (80% death). When the NPs were further complexated with SiRNA against luciferase they exhibited a strong PCI effect at 810 nm, reducing the MCF7 intracellular luciferase luminescence by 64%.

### 5.2. PCI for the Delivery of mRNA

The use of mRNA for therapeutic and vaccination approaches has a very great potential [176]. Although the incorporation of modified bases into mRNA molecules have reduced the susceptibility for mRNA inactivation by RNases [177], mRNA stability is still an issue, and the lack of efficient and specific methods for in vivo delivery of mRNA molecules is still a major barrier for the realization of their therapeutic potential. Most mRNA delivery methods depend on endocytic uptake, and, given the instability of mRNA, it is very important to be able to release the mRNA molecules from endocytic vesicles before they are degraded [178]. PCI is an ideal technology for achieving this, since endosomal release can be induced instantly by illumination shortly after mRNA uptake. The first study reporting PCI-mediated mRNA delivery in vitro was published by Bøe et al. [179], using PEI as a delivery vehicle for the mRNA. Later experiments by the same group showed efficient PCI-induced mRNA delivery also with a poly-l-arginine delivery vehicle. Since this vehicle has no endosomolytic properties in itself, in this case PCI could be used for turning mRNA expression on, against a background of virtually no expression without PCI. PCI-mediated mRNA delivery was also investigated with various PAMAM-based formulations, but while such formulations were effective for PCI-mediated siRNA delivery, they did not work with mRNA [167]. In vivo, PCI-mediated mRNA delivery using delivery vehicles have so far largely been unsuccessful, but very promising results have recently been obtained with PCI-mediated delivery of various types of “naked” mRNA molecules (Høgset et al., manuscript in preparation).

## 6. PCI for Gene Delivery

### 6.1. PCI for Plasmid Delivery

DNA plasmids have many potential very interesting applications, such as the use in vaccination, cancer therapy and therapies for congenital diseases. Despite many years of research, the potential of plasmid-based medical applications has not yet been realized, with only two products on the market, Neovasculogen™ (approved in Russia for the treatment of atherosclerotic peripheral arterial disease) and Collategene^®^ (approved in Japan, also for the treatment of peripheral arterial disease). As for mRNA-based approaches proper in vivo delivery is probably the most important hurdle for realization of the potential of plasmid-based therapies and vaccination. For vaccination purposes direct intramuscular injection may work well in animals, and physical technologies such as “gene gun” and electroporation have shown promise, but such technologies are far from optimal for wide-spread use in humans. The promising results seen with plasmid-based DNA vaccination in animal studies have so far unfortunately not been transferable to humans, although several plasmid based human vaccination studies are on-going (reviewed in Hobernik and Bros [180] and Liu [181]). Many plasmid delivery technologies mainly rely on different types of delivery vehicles, and for such systems, endosomal escape represents a very important delivery barrier. In a very interesting study de Bruin et al. [80] investigated the kinetics of PCI-mediated endosomal release for delivered DNA, showing that under the conditions used both PEI- and PLL-complexed DNA was released within seconds after the start of illumination.

There is an extensive literature showing that PCI can enhance plasmid delivery by a variety of vehicle systems as reviewed in the following.

In vitro, PCI gives a strong enhancement of plasmid delivery mediated by cationic polymers, with good effects shown with, e.g., cationic poly-amino acids [159], PEI [85,182] and PAMAM dendrimers [183]. While some such vehicles (e.g., PEI) have an inherent endosomolytic function, others, like poly-L-lysine, have no such properties [184] and with the latter type of vehicles PCI can essentially turn plasmid transfection on, against a background of no transfection. This can be very important in in vivo situations where it is desirable with a strictly site-confined delivery.

Gaware et al. [185] tethered the PS 5-(p-aminophenyl)-10, 15, 20-triphenylporphyrin to 3, 6-di-O-tert-butyldimethylsilyl-chitosan which assembled into NPs with a core of π-stacked PSs. These NPs could then unfold in the lipophilic environment of cell membranes where the PSs could be inserted. The PCI efficacy for gene transfection (EGFP) was tested on HCT116/LUC human colon carcinoma cells and a notable increase of EGFP-positive cells (from 0.2% to 20%) was observed after PCI application.

In several very interesting articles Kataoka and co-workers have described the construction of nanoparticles for PCI-mediated DNA delivery where the DNA is packaged inside an envelope of a dendrimeric photosensitiser so that both the DNA and the photosensitiser will be delivered to the cell in one package [186]. With this system DNA delivery could be enhanced > 100 times upon illumination, and the system worked well also for in vivo delivery of DNA to the eye in mice. The same group also developed the dendrimeric photosensitiser with PEGylated polyplexes, in a system exhibiting an even higher degree of light induction of transfection [187].

A dendrimeric photosensitiser system for PCI-mediated plasmid delivery has also been developed by Shieh and co-workers [183], where a PAMAM dendrimer photosensitiser conjugate was used as a DNA complexing agent, exhibiting good transfection upon illumination. In later studies, several new nanoparticulate systems for PCI-mediated nucleic acid delivery have been described, such as a helical polypeptide based system [188], and a poly-amino acid based system for co-delivery of plasmids and drugs [189].

Based on the initial work with several of the commonly used plasmid delivery vehicles several groups have developed PCI delivery with more advanced delivery systems. For example, the use of vehicles designed for receptor-mediated targeted cellular uptake has given very good results, using e.g., EGF [190,191] or transferrin [95] as receptor ligands. Thus, in some of these studies a PCI-induced transfection enhancement of > 100 times has been observed.

Pheophorbide A as a PCI-PS induced only a minor effect on transfection efficacy, at least when the plasmid is complexed with asymetric polyplex-nanoparticles based on PEG-PCL-PEI copolymers constructed by solvent-injection [192]. In another study by Cho et al. [193], pheophorbide A (PS)-loaded, thiol-degradable polymeric complexes with a CMV-promoter/luciferase DNA plasmid were constructed. The polyplexes were able to escape the endocytic vesicles due to the “proton sponge effect” induction by the polycationic backbone, and consequently intracellular GSH reduction of the disulfide bonds led to a further release of pheophorbide A and DNA in the cytosol. The polyplexes showed a further irradiation enhancement of MDA-MB-231 cell transfection, which was due to a PCI effect but also, according to the authors, due to ROS-induced activation of the CMV promoter.

Han et al. [194] complexed DNA encoding tumor necrosis factor related apoptosis inducing ligand (TRAIL) with branched polyethylenimine (bPEI), and treated human mesenchymal stem cells (hMSCs) in the presence and absence of pheophorbide A as the PS. This treatment profoundly enhanced the capacity of hMSCs to secrete TRAIL as was shown in co-culture of hMSCs and MIA PaCa-2 pancreatic carcinoma cells, where the photochemical treatment mediated a significant TRAIL-induced cytotoxicity to the MIA PaCa-2 cells. The authors further evaluated the effect in vivo, on a MIA PaCa-2 mouse xenograft model by injecting PCI activated hMSCs intravenously, instigating a complete tumour growth inhibition and even tumour shrinkage within a 15-day follow-up.

Wang et al. [195], designed a ROS-degradable polycation based on tioketal-crosslinked polyethylenimine (TK-PEI) to complexate with p53 or luciferase encoding plasmid DNA, while hyaluronic acid modified with pheophytin a, a cholophyll molecule lacking central Mg^2+^ acting as a PS, was coated on the nanocomplexes for colloidal stability enhancement and photodynamic action. PCI was found to considerably enhance the transfection efficiency (~5×) of luciferase in B16F10 mouse skin melanoma, HeLa and COS-7 monkey, fibroblast like cells. The increase in transfection efficiency due to the “dual unwrapping” (PCI and NP-collapse) by light induced ROS, was also confirmed in vivo, following intratumoral injection to melanoma-bearing mice. The PCI-assisted transfection of p53 DNA exerted profound anticancer effects both in vitro and in vivo in B16F10 melanoma models.

As is the case with siRNA (see Section 5 above) the PCI effect on lipoplex-mediated DNA delivery is more variable, seemingly more dependent on the exact structure of the lipoplex, the experimental variables, and probably also on the cell line [196].

From the DNA transfection studies reviewed herein, it is notable that the most frequent plasmid DNAs used were those of GFP and luciferase (Luc). These studies primarily aimed at showing the impact of the combination of the engineered nanocarriers and PCI on transfection without any therapeutic implications. However, several reports also describes enhanced effects of therapeutic genes after PCI-mediated gene delivery, e.g., for genes encoding pro-drug converting enzymes [197,198], p53 [199,200], and tumour necrosis factor-related apoptosis-inducing ligand (TRAIL) [194].

PCI with a PEI-based delivery vehicle has also been applied in vivo for gene therapy in a mouse model of head and neck squameous cell carcinomas deficient in active p53. The photosensitizer AlPcS_2a_ and a plasmid encoding p53 were delivered as weekly intratumoural injections and caused the induction of apoptosis leading to dramatic tumour regression in all the PCI-treated animals [201]. This was not seen in any of the control groups, clearly showing that the PCI-mediated enhancement of plasmid delivery was essential for achieving the desired therapeutic effect of the gene therapy.

### 6.2. Viral Gene Delivery Systems

Viral systems are commonly used for the delivery of therapeutic genes to cells, both for transient expression, and with the aim of stable integration of a transgene into the genome of a target cell. As compared to non-viral delivery vehicles viral systems are usually more efficient, among other things due to enhanced uptake into cells, and inherent functions for endosomal escape and nuclear trafficking. However, many viral systems have severe safety concerns, and in general it would be very advantageous to be able to target virus-mediated gene delivery to the desired sites in the body, and also to use as low doses of virus as possible to diminish production problems, safety concerns and the induction of unwanted immune reactions. For many virus types endocytosis is an important mechanism for uptake into the cell, and although most viruses as part of their life cycle have endogenous mechanisms for endosomal escape of their genetic material, these mechanisms may not always be fully efficient. Thus, the use of PCI for enhancing virus-mediated gene delivery has been explored with two commonly used viral gene delivery systems where endocytosis is known to play an important role for cellular uptake. Adenovirus serotype 5 (Ad5) is usually taken up into the cell by endocytosis after binding the coxsackievirus and adenovirus receptor (CAR) receptor, and it was generally believed that after uptake most viral particles escape from the endosomes [202,203]. It was therefore somewhat surprising that PCI could strongly enhance adenovirus-mediated transduction, with an enhancement of >20 times achieved by the employment of PCI [204,205]. In the same study dose response experiments showed that the transduction-enhancing effect of PCI was especially good at low virus doses, something that may be very valuable in the clinical use of adenovirus mediated therapies. Uptake by the CAR receptor in many cases represents a limitation for Ad5-mediated gene delivery, since many potentially important target cells (e.g., airway epithelial cells, smooth muscle cells and most cancer cells) have low expression of this receptor. However, uptake of Ad5 in CAR-negative cells can be enhanced un-specifically by coating the virus particles by cationic polymers [206,207], or, specifically, by furnishing the virus surface with a ligand for a specific receptor on the target cell surface. PCI should be well suited both for enhancing the efficacy and the site-specificity of Ad5 mediated gene transduction with such methods, and in several studies a strong PCI-induced enhancement has been achieved for both polycation-mediated [208,209] and for receptor-mediated [210] uptake of Ad5 virus particles.

Adenovirus associated virus (AAV) is a commonly used vector for permanent transduction of cells: AAV is usually taken into the cell by endocytosis and for several types of AAV endosomal trafficking has been described to be a limiting factor in the transduction process [211,212]. Thus, a study was performed using PCI for enhancing AAV-mediated gene delivery to glioblastoma cells, and it was shown that PCI could enhance transduction also for this virus type [213].

## 7. Considerations Regarding the Use of PCI to Enhance the Therapeutic Effect of Nucleic Acids

The PCI-induced enhancement of nucleic acid delivery across a wide range of nucleic acid and delivery vehicle type confirms the central role of endosomal escape as a barrier for intracellular nucleic acid delivery. Even for several delivery systems believed to be quite efficient in endosomal escape, such as adenovirus, and lipidic vehicles such as lipofectamine significant improvement could be induced by PCI, strongly indicating endosomal escape as an important limitation also for such vehicles. Both for adenoviral and for lipidic delivery vehicles there however seem to by substantial differences in the PCI effect between different cell types [196,214], indicating differences in endocytic trafficking and the processing of such vehicles in different cell types.

Many polymeric delivery vehicles (e.g., PEI) are believed to effect endosomal escape through a so-called “proton sponge” effect [215], where protons are captured by the vehicles leading to endosomal swelling and rupture of the endosomes. For this mechanism to be effective it is necessary with a minimum amount of vehicle inside the endosomes to induce the necessary endosomal swelling. While this may be easy to achieve in vitro, it is much more difficult in vivo, especially since such vehicles often induce toxic reactions strongly limiting the amount of vehicle that can be injected. It is therefore very interesting that PCI is able to enhance nucleic acid delivery with such vehicles in amounts far below what is needed for the induction of endosomal escape by the “proton sponge” effect [214,216], potentially making it possible to use the vehicles also for in vivo applications where vehicle toxicity would normally be of concern.

Polycations can be used for effecting nucleic acid uptake by conferring a positive charge to the negatively charged nucleic acids so that a polycation/nucleic acid complex can be taken up after electrostatic interaction with the negatively charged cell surface. This is a general uptake mechanism that works very well in combination with PCI with many types of nucleic acids ranging from PNAs conjugated to positively charged amino acids [163,217] thorough poly-amino acid mediated delivery of plasmids and mRNA [159,166], to adenovirus coated with polycations [208]. To the extent that the employed polycations are not able to induce a “proton sponge” effect, such systems will often have very low gene delivery efficiency on their own, meaning that PCI can be used to literally turn delivery on, with enhancement factors up to >100 times being observed [85,190]. Thus, such systems could be very useful in situations where a strict spatio-temporal control over the delivery is desired.

For many types of delivery vehicles PCI seems to have the same positive effect across all the different nucleic acid cargos tested, but this is not always the case, e.g., for PAMAM dendrimers PCI seems to work well for siRNA, but not for mRNA delivery [167]. While one might expect the PCI effect on endosomal escape to be the same irrespective of the nucleic acid cargo, such difference may, e.g., be caused by different sensitivities of the cargo to degradation inside endocytic vesicles, e.g., making the timing of endosomal release an important parameter. Thus, while for mRNA it seems very important to induce endosomal release rapidly after uptake (Høgset et al., manuscript in preparation), e.g., for plasmids this seems comparatively less critical [214].

When using the fimaporfin (TPCS_2a_) photosensitizer [51], which is currently being developed in clinical studies [106], the photosensitizer molecule is added as a separate entity, not being chemically or physically attached to the other components of the delivery system. For many applications it could however seem advantageous to have the photosensitiser attached to the nucleic acid cargo, the delivery vehicle or both. Such systems have been developed both for oligonucleotides [162,163] and for plasmid [186] delivery, and has been shown to work well. While there are obvious advantages of having all components in one cargo, it however also makes the synthesis and formulation processes more complicated, and for some covalent systems it may also be a problem to achieve optimal doses of all components in the system at the same time. Thus, using PCI with small molecule photosensitizer like fimaporfin constitutes a flexible and efficient system for nucleic acid delivery. PCI can be used for enhancing the delivery of nucleic acids administered both systemically and locally, but given that systemic delivery of nucleic acids to other organs than the liver is notoriously difficult and that PCI in its nature is a technology for enhancing delivery locally, local administration of the nucleic acid and the photosensitisers is a logical place to start in the in vivo exploration of PCI mediated nucleic acid delivery. Thus, in on-going animal experiments very promising results have been achieved using PCI for mRNA delivery to skin and to tumours (Høgset et al., manuscript in preparation), opening very interesting possibilities for the use of PCI in the development of therapies based on mRNA.

## 8. PCI in Antitumor Immunity and Cancer Vaccination

Successful therapeutic cancer vaccination is dependent on a robust priming and stimulation of cancer-specific CD8^+^ cytotoxic T lymphocytes (CTLs) as these immune cells are the most efficient tumor cell killers. A critical step before vaccine-induced CTL activation is the endocytosis of the antigen into antigen-presenting cells (APC), of which dendritic cells (DCs) are the most professionals. Subsequently, cytosolic antigen processing by the major histocompatibility complex class I molecules (MHC I) presentation machinery and finally loading of the antigen onto MHC I take place, resulting in cross-presentation of the antigen to CD8^+^ CTLs. However, intracellular uptake of peptide/protein-based antigens by means of endocytosis mostly results in transportation of the antigen(s) into late endosomes and lysosomes for enzymatic degradation, which results in MHC II presentation and activation of CD4^+^ T lymphocytes. Although CD4^+^ T-cells plays important roles in anti-tumor immunity, there is solid pre-clinical evidence that triggering CD8^+^ CTLs is important for the elimination of tumor cells [218]. Thus, to circumvent the dominance of the MHC class-II pathway, there is a need for strategies that enable enhanced endosomal escape of antigens for improved MHC class-I cross-presentation and triggering of effector and memory antigen-specific CD8^+^ CTLs responses.

PCI mediated cytosolic release of a synthetic peptides derived from mutated p21 ras was already demonstrated in the first article describing the PCI technology [48]. Recently, PCI has been demonstrated in different preclinical studies to be a promising method for enhanced endosomal escape and cytosolic delivery of both protein and synthetic short or long peptide-based antigens. As proof of concept, PCI-enhanced prophylactic cancer vaccination was demonstrated by using the ovalbumin (OVA) protein in the B16F10-OVA in vivo model [219]. The PCI photosensitizer disulfonated tetraphenyl chlorin (TPCS2a/fimaporfin) was administered together with OVA intradermally (i.d.) to C56BL/6 mice. Eighteen hrs after i.d. injection of the vaccine, the skin was exposed with blue light (λmax ≈ 430 nm) for activation of the PCI-photosensitizer and vaccine antigen. Strong activation of OVA-specific CD8+ CTLs, adoptively transferred from T cell receptor transgenic mice, and expression of intracellular IFN-γ and secretion of IL-2 was measured [219]. In a succeeding study, dermal PCI of OVA also resulted in strong therapeutic cancer vaccination effects, with activation, proliferation, and IFN-γ production of CTLs, which infiltrated B16F10-OVA tumors and inhibited tumour growth probably by caspase-3-dependent apoptosis. TPCS2a and OVA were shown to co-localize in bone marrow-derived DCs prior to PCI-induced cytosolic delivery of antigen [220]. The PCI-enhanced anti-cancer vaccine effect was dependent on trypsin- and caspase-like proteasome activity and independent on MHC II, MyD88 and TLR4 signalling [220]. The independency of MHC II and CD4^+^ T cells was recently validated in MHC class II- or CD4^+^ T-cell-deficient mice [221].

Mechanistically, direct evidence of strong PCI-enhanced loading of antigen (OVA257–264 peptide SIINFEKL) onto MHC I was recently revealed in both immature BMDCs and the B6 macrophage cell line using the anti-H-2Kb-SIINFEKL, which is a mAb that detects MHC class I molecule Kb bound to the peptide SIINFEKL [222]. Also in this study, PCI-enhanced cytosolic delivery of antigen (SIINFEKL) was shown. Furthermore, it was demonstrated that the PCI technology is able to prime naïve endogenous CD8+ CTLs cells in vivo by utilizing the clinically relevant peptide antigens HPV 16 E7 protein (HPV43–78 peptide) and the melanoma-derived TRP-2180-188 peptide, while these antigens alone were ineffective [222]. Analysis of spleen cells revealed cancer antigen-specific IFN-γ effector cytokine production, which indicates that PCI vaccination results in activation of functional CD8^+^ effector T cells.

The importance of anti-tumour immunity and curative effects after PDT and PCI of bleomycin (a study not designed as a cancer vaccine per se) was shown in the CT26.CL25 tumor model, that constitutively express the bacterial β-galactosidase antigen [223]. PCI of bleomycin (AlPcS_2a_ + bleomycin + light) was found to instigate synergistic inhibition of tumor progression as compared to the sum of the individual treatments in immune competent (thymic) mice. The same effects were obtained with PDT only, however, it was necessary to give higher light doses, indicating that bleomycin may play a role in the development of anti-tumor immunity after PCI. Strikingly, in athymic nude mice lacking mature T-cells, no curative effects were achieved with neither PDT nor PCI. Of relevance, PDT has for many years been reported to trigger a stimulation of the innate and adaptive immunity important for both tumor cell eradication and long term memory and tumor control in preclinical models [224].

In conclusion, PCI is demonstrated preclinically to enhance MHCI crosspresentation and to be a very promising method for strong enhancement of cancer vaccine efficacy [219,220,222]. PCI-enhanced vaccination has also recently been validated in a Phase I study on healthy volunteers, with the objective to assess safety, tolerability and immune response after PCI of human papilloma virus E7 (HPV E7) and keyhole limpet hemocyanin (KLH) antigens (ClinicalTrials.gov Identifier: NCT02947854). This study (Høgset et al. manuscript in preparation) confirmed preclinical results (unpublished data) showing that PCI significant enhances cellular immune responses to HPV peptide antigens.

## 9. PCI Approaches to Treatment of Brain Tumors

### 9.1. Background

Even with substantial improvements in conventional treatments consisting of surgery, radiation therapy and chemotherapy, the prognosis for patients with malignant gliomas (GBM) has not improved significantly over the past four decades. This is due to the aggressive infiltrating nature of malignant gliomas: many glioma cells have already infiltrated 2–3 cm into the surrounding normal brain at the time of cytoreductive bulk tumor resection. These infiltrative tumor cells are well embedded in brain tissues, supplied with nutrients and oxygen by the normal brain vasculature and consequently, also protected by the blood–brain barrier (BBB). This barrier plays a pivotal role by preventing harmful substances from entering the brain’s microenvironment. Unfortunately, few anti-cancer drugs can effectively cross this barrier to target these infiltrating tumor cells. Failure to eradicate infiltrating glioma cells inevitably results in tumor recurrence and further treatments are usually palliative in scope. Tumor resection is usually the first modality employed in the treatment of gliomas. Although gross tumor resection as defined by a negative post-operative MRI can be obtained in a majority of cases with current available techniques [225,226], patients continue to relapse. Since 80%‒90% of GBMs recur within 2 cm of the resection margin [227,228], a reasonable starting point for improving the prognosis of GBM patients would be the development of improved local therapies capable of eradicating glioma cells in the tumor resection margin and brain-adjacent-to-tumor (BAT) region.

### 9.2. Potential Uses of PCI for the Treatment of Brain Tumors

#### 9.2.1. Bypassing the BBB

PCI, by its nature, is site specific and is localized to the area receiving sufficient light energy. PCI therefore applied in the tumor resection cavity would have a twofold effect; localized opening of the BBB would for the first greatly enhance the delivery of therapeutic agents and secondly would increase the efficacy of the delivered agent. The ability of PCI to enhance the efficacy of anti-tumor agents in general is covered in detail in other portions of this chapter.

Local disruption of the BBB for drug delivery into the brain has been accomplished using a number of approaches including laser-based techniques such as PDT and PCI [229,230]. The light-based approaches are appealing due to the highly localized nature of the BBB disruption which can be limited to the vicinity of the post-operative tumor resection cavity. The BBB is only disrupted at sites subjected to sufficient laser power densities required to activate the photosensitizer. Equally important are observations showing that these highly focused approaches do not cause permanent damage to the BBB, as long as incident power densities remain below threshold levels. In particular the laser-based modalities cause the localized area of the BBB to remain open for relatively long periods of time (days) thus facilitating multi-fractionated drug delivery. In contrast, the use of hyperosmolar solutions opens the BBB globally for a relatively short time and has proven unsuccessful in clinical trials both for its lack of efficacy as well as causing damage to normal brain [231].

**Figure 5:**
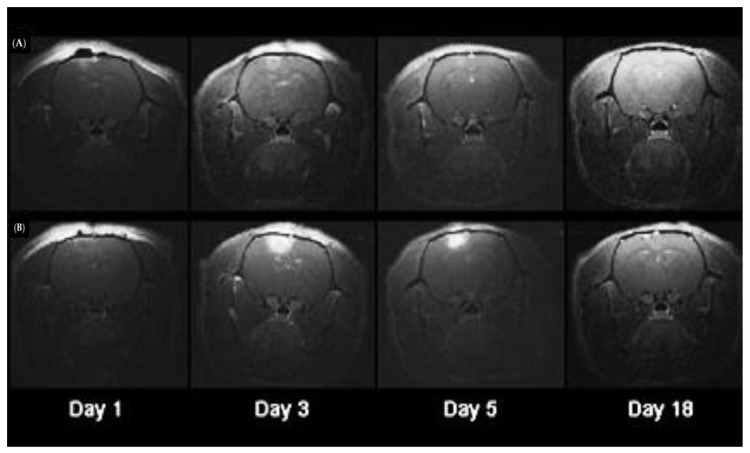
Comparison of T1-weighted post contrast MRI scans after PDT and PCI treatment. (**A**) The PDT treated animal received a light fluence of 1 J; (**B**) the PCI treated animal received a light fluence of 1 J and an i.p. injection of Cl p at a concentration of 1:100. Both animals were scanned on days 1, 3, 5 and 18 after treatment. All T1 post contrast images were taken 15 min following i.p. contrast injection.

Localized BBB opening via PCI-mediated delivery of *Clostridium perfringens* epsilon prototoxin (Clp) was investigated by Hirschberg and coworkers in Fischer rats [230]. The rationale for using Clp is due to the ability of active toxin to cause widespread but reversible opening of the BBB [232]. Following systemic administration, Clp prototoxin is converted to fully active toxin by proteolytic cleavage. It was hypothesized that the biological effects of suboptimal doses of Clp epsilon prototoxin on the BBB will be potentiated by PCI, but only in areas of the brain exposed to adequate light fluences. The results from this study demonstrated that Clp-PCI was capable of causing localized BBB disruption at very low light fluences (1 J/cm^2^) and sub-threshold Clp concentrations as shown in Figure 5. Of particular interest was the time duration and evolution of the Clp-PCI BBB disruption since this represents the therapeutic window for drug delivery. Based on an analysis of MR images, enhancement volumes were observed to peak three days following Clp-PCI suggestive of maximum BBB opening at that time (Figure 5). Thereafter, contrast volumes were observed to decrease and by day 11 only trace amounts of contrast were observed.

**Figure 6:**
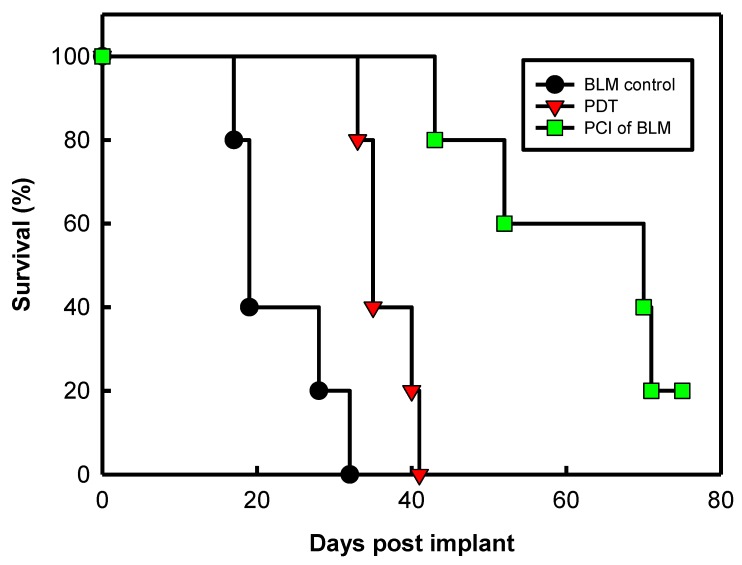
Treatment protocol and Kaplan–Meier survival of tumor cell implanted animals. All animals received 1 x 10^4^ cells i.c. Three groups were followed: Control (BLM (bleomycin) 8 mg/kg), PDT-BLM (AlPcS_2a_ 1J), Clp-PCI BLM (AlPcS_2a_, Clp, 1 J); BLM 8 mg/kg was injected i.p. twice daily for 3 days after light exposure. Reproduced from Hirschberg et al. [225].

In an in vivo study, using an orthotopic brain tumor model consisting of F98 glioma cells in Fischer rats, newly implanted tumor cells were used to mimic the characteristics of infiltrating cells remaining in the resection margin usually found following surgical removal of bulk tumor [230]. AlPcs_2a_ PDT or Clp-PCI localized BBB opening was performed 24 h after cell inoculation. This is an insufficient time to allow for the development of bulk tumor and BBB degradation, but long enough for the cells to form small, sequestered, micro-clusters which are protected by an intact BBB. The survival of animals implanted with F98 tumor cells, as shown in Figure 6, was significantly extended following BLM chemotherapy with Clp-PCI mediated BBB opening compared to controls that received chemo- or PDT-chemotherapy alone. Chemotherapy (BLM) was administered during and after the light treatment.

The second factor limiting the efficacy of BLM chemotherapy towards glioma cells, i.e., endosomal entrapment, was also examined in in vitro experiments employing multicell tumor spheroids formed from human or rat glioma cells [233]. An advantage of spheroid cultures is that their micro-environment more closely mimics the in vivo situation than monolayer cultures and therefore the cells are likely similar to that encountered in tumor cells in situ. The inhibitory effects on spheroid growth by BLM-PCI were compared to the effects of BLM alone. PCI greatly enhanced the effects of the drug, and the effects of PCI with 0.1 g/mL BLM were equivalent to those observed at 5 g/mL of drug alone. Similar enhancement of the efficacy of BLM by PCI was obtained on rat glioma cells by Gederaas et al. using the clinically approved photosensitizer meso-tetraphenyl chlorin disulphonate (TPCS_2a_; Amphinex) [234]. This dramatic increase in drug efficacy would allow much lower systemic drug doses to be administered to patients to obtain similar or improved effects compared to those obtained by drug alone. This in turn could eliminate the severe cognitive dysfunction, often referred to as “chemobrain”, a condition that can persist long after the cessation of treatment in as many as 75% of brain cancer patients.

#### 9.2.2. Macrophages as Delivery Vectors for PCI Mediated Chemotherapy

**Figure 7:**
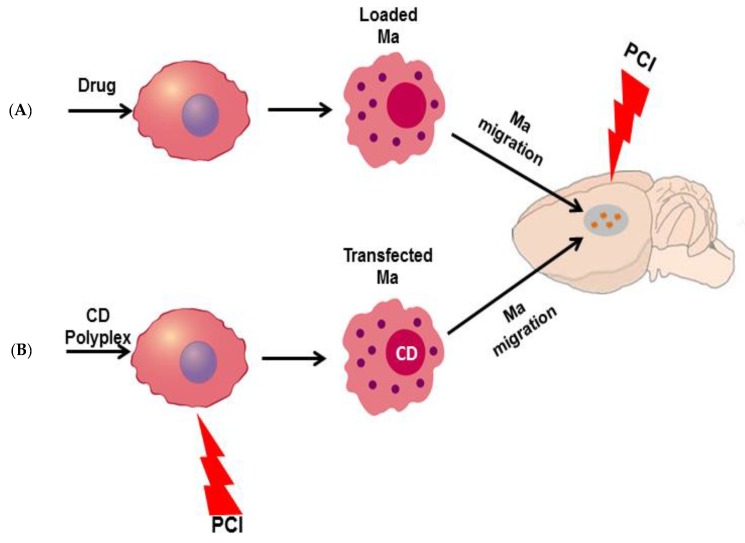
PCI enhanced macrophage drug delivery to tumors by 2 alternative pathways. (**A**) Incubation of Ma with drug loads Ma, Ma infiltrates tumor, the drug is released by PCI. (**B**) Ma are PCI transfected with a gene that converts a nontoxic prodrug to a toxic drug. Ma infiltrates tumor, prodrug administered, converted toxic drug is released.

The passive accumulation of nanoparticles (NP) in tumors via the enhanced permeability and retention (EPR) effect has been considered one of the main advantages of this approach. Although numerous studies have verified the EPR effect in rodent tumor models, clinical results have been disappointing and suggest that NP as delivery vehicles are ineffective in humans [235,236]. Macrophages (Ma) have been used in targeted delivery approaches of a variety of anticancer agents including nanoparticles, chemotherapeutics, proteins, suicide genes and viruses [237]. Ma actively migrate to and infiltrate tumors via gradients of cytokines, growth factors and chemokines despite elevated interstitial pressures and stromal barriers encountered in brain as well as other types of tumors. Drugs can be transported to tumors using Ma as vectors by two different approaches: (1) Ma are directly loaded with drug ex vivo (Figure 7A), or (2) are ex vivo PCI enhanced transfected with a suicide gene (Figure 7B). In either case the loaded/transfected Ma are reinjected into the patient, infiltrate the tumor, release the drug, followed by PCI drug effect enhancement. In either approach the released drug is taken up by the tumor cells, the so-called bystander effect.

##### Drug Release from Loaded Ma

**Figure 8:**
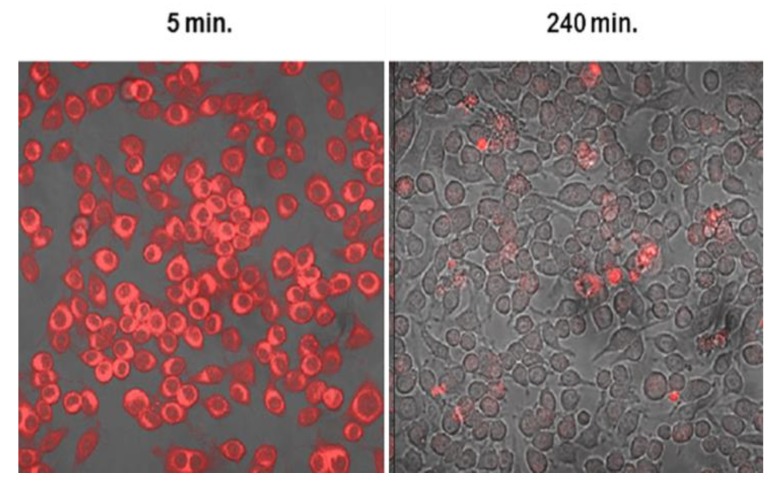
Drug release from DOX loaded Ma over time. Ma were incubated with 100 µg/mL DOX for 2 min, followed by double wash to remove non-incorporated drug. Images were captured with fluorescence microscopy 5 min 240 min later. DOX is shown by red reflectance dispersed throughout the cells.

Drug delivery via loaded Ma has been demonstrated in vitro on hybrid 3D glioma/Ma hybrid spheroids. Employing hybrid spheroids, consisting of mixtures of rat glioma cells and doxorubicin-loaded murine Ma, Shin et al. [238] showed that Ma were capable of incorporating and releasing un-degraded doxorubicin as seen in Figure 8. Initially (5 min) the Ma contained high concentrations of DOX (Figure 8). After 4 h of incubation in drug free culture medium the Ma contained limited or no drug (Figure 8).

The results also clearly demonstrated that AlPcs_2a_-PCI could greatly enhance the efficacy of released drug from the subpopulation of Ma contained in Gliom-Ma hybrid spheroids. The growth of the hybrid spheroids was synergistically reduced or completely inhibited by PCI compared to either PDT or Ma released drug acting as single treatments. This PCI enhanced drug efficacy would compensate for a limited number of drug-loaded Ma infiltrating into the tumor, in turn limiting the amount of active drug delivered.

##### Ma-Mediated PCI-Enhanced Gene-Directed Enzyme Prodrug Therapy

Gene-directed enzyme prodrug therapy, also called suicide gene therapy, involves transfection into tumor cells of non-mammalian genes encoding enzymes that convert nontoxic pro-drugs into toxic metabolites capable of inhibiting nucleic acid synthesis. Among several approaches currently being developed is the induction of cytosine deaminase (CD), an enzyme which converts the relatively nontoxic antifungal agent 5-fluorocytosine (5-FC) into the potent antimetabolite 5-fluorouracil (5-FU). This system has been previously studied for a variety of tumor types. Importantly, the bystander effect, where activated drug is exported from the transfected cells into the tumor microenvironment, plays an important role by inhibiting growth of adjacent tumor cells. The difficulty of transfecting a sufficient number of tumor cells in vivo has led to limited therapeutic success. Christie et al. [239] proposed the basic concept of ex vivo non-viral transfection of the CD gene into Ma using PCI to increase transfection efficiency (Figure 7B). The tumor infiltrating Ma carrying the prodrug activating gene (CD) will upon exposure to the pro-drug (5-FC) produce and export the active drug (5-FU) into the tumor microenvironment, the so-called bystander effect. Previous in vitro experiments have demonstrated that only a small subset of CD gene transfected cells, infiltrating into three-dimensional tumor spheroids were capable of completely inhibiting their growth upon exposure to 5-FC [198,240]. Although Mas have proven difficult to transfect with non-viral vectors the use of PCI increased the transfection rate of the CD gene from 6% to 35% (unpublished observation) which via the bystander effect should be adequate for effective treatment.

PDT/PCI, has been shown to potentiate the efficacy of chemotherapy. It was therefore hypothesized that AlPcS_2a_-PDT/PCI should be capable of increasing the efficacy of the compounds delivered by drug loaded Ma or converted from prodrug by CD gene transfected cells, thus in effect reducing the number of transfected cells required for effective therapy (Figure 3A,B). Christie et al. showed that the efficacy of 5-FU converted from 5-FC by CD positive cells interacted in a synergistic manner over a range of prodrug concentration and tumor to transfected cell ratios [241]. The degree of synergy was significant regardless if PDT/PCI treatment was given before or after 5-FC administration although the highest degree of interaction was observed when light treatment was delivered prior to prodrug exposure.

#### 9.2.3. Localized Drug Delivery

An alternative method of bypassing the BBB to those already illustrated is local controlled release of chemotherapeutic agents. Fibrin glue (FG), a mixture of thrombin and fibrinogen, loaded with drugs has been studied as a localized controlled release vehicle. In the simplest formulation exogenous drugs are added to one of the components before addition of the other allowing the drug to be distributed throughout the solution before cross-linking occurs. While fibrin glue has shown promise in delivering a slow release vehicle of drugs, the quantity and release kinetics attainable has some limitations.

**Figure 9:**
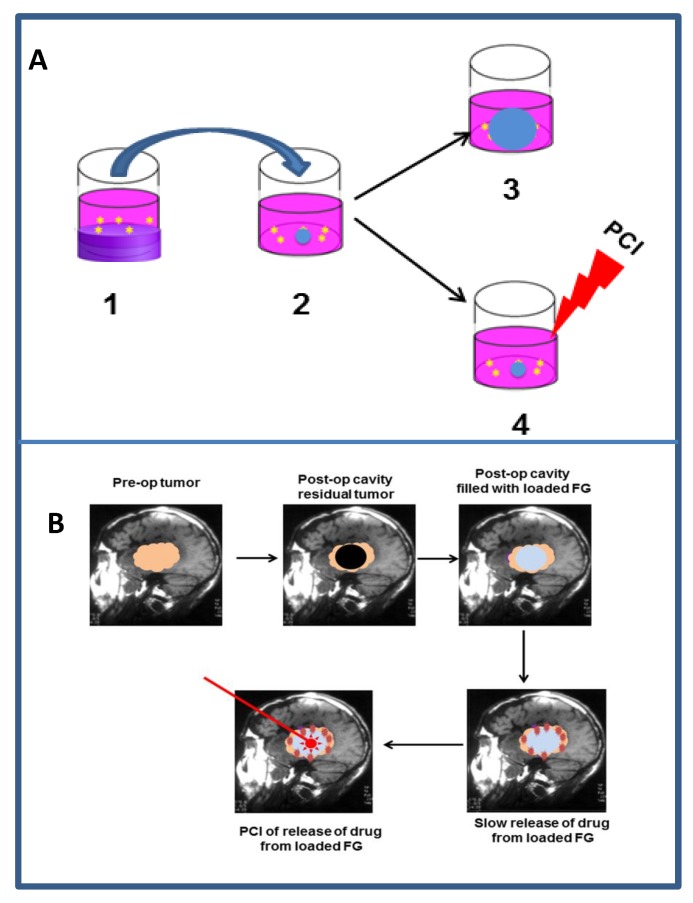
(**A**) Drug release from fibrin glue. (1) Fibrin glue (FG) layers formed with incorporated drug overlaid with culture medium. (2) Supernatant harvested at different times from drug containing FG layers co-cultured with spheroids formed from glioma cells pre cultured with photosensitizer in the (3) absence or (4) presence of light treatment (PCI). Spheroid growth was monitored for 14 days. (**B**) Proposed clinical translation of FG-PCI protocol. Following tumor resection, the resection cavity is filled with drug/photosensitizer loaded FG. The FG slowly dissolves releasing the drug. Light treatment done through a temporarily surgically placed optical fiber positioned in the center of FG insert.

The combination of local intra-cavity FG slow release drug delivery combined with PCI therefore has the potential of bypassing the BBB and allowing increased chemotherapeutic efficacy. In vitro proof of principle experiments using the drugs bleomycin (BLM) or doxorubicin (DOX) have been carried out using an experimental set up shown in Figure 9A (Nguyen et al. submitted).

The in vitro results of these experiments demonstrated that at the BLM or DOX concentrations used in these experiments, to load the FG, spheroid growth was not significantly influenced by the BLM or DOX that was released from the FG gels. In contrast, spheroid growth was significantly inhibited or completely suppressed by PCI of released drug from either FG-BLM or FG-DOX. The release of BLM from FG was more rapid than that seen for DOX. AlPcS_2a_, released from FG, together with light treatment could also provide for effective photodynamic therapy (Figure 9B).

#### 9.2.4. Light Delivery to the Resection Cavity

Since the efficacy of PDT depends on the ability to deliver adequate fluences to malignant cells in the resection margin, careful consideration must be given to the light delivery technique. Short-term intraoperative light delivery is unlikely to eradicate tumor cells deep in the margin due to the inability to deliver threshold fluences in a reasonable time period.

**Figure 10:**
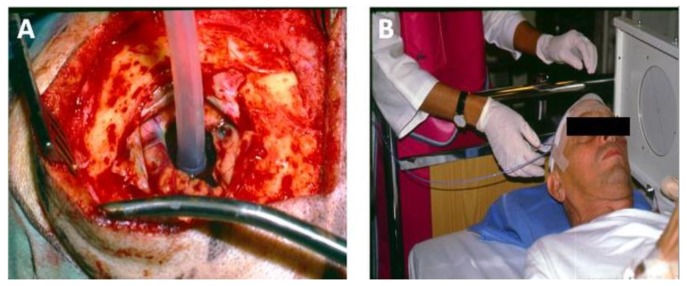
PDT treatment of a glioblastoma patient. (**A**) Implantation of an indwelling balloon catheter for use as a radiation or light applicator. After closure of the operative field the catheter is brought out through the skin and remains in place for several days. (**B**) A patient receiving after-loading brachytherapy [242]. The same applicator can be used for PCI.

A solution to the problem of inadequate light delivery is to insert an indwelling applicator into the resection cavity (Figure 10A), remaining in place for a period of days. This would allow for radiation or light delivery over extended time periods as shown Figure 10B; a patient receiving after-loaded brachytherapy [242]. Such an applicator would also facilitate investigation of other light delivery schemes, such as fractionation and long-term repeated PDT treatments suited to such treatment regimens [243,244]. A future alternative may be to replace photons with protons in glioblastoma treatment since protons have been found to induce singlet oxygen when hitting photosensitizers [245].

#### 9.2.5. Metronomic PCI

Due to the rapid attenuation of light in tissue the ultra-low-intensity laser light irradiance that would be found at cm depths in the BAT would prove inadequate if delivered over the relatively short treatment periods, measured in minutes, usually employed with PCI. Shin et al. have recently shown that BLM-PCI could effectively cause significant spheroid growth inhibition with the delivery of extremely low light irradiance rates (W/cm^2^ irradiance level, usually described as metronomic treatment [246]) if delivered over a period of time measured in hours [247]. Those findings suggested that effective implementation of metronomic PCI can deliver adequate drug efficacy at depths necessary to reach infiltrating glioma cells in the surgical resection cavity wall. A future alternative may be to replace photons with protons or ultrasound in glioblastoma treatment since protons and ultrasound have been found to induce singlet oxygen and therapeutic effects when hitting the photosensitizers [241,245,248,249].

#### 9.2.6. PCI for Treatment of Brain Tumors-Conclusion

To date, no clinical trials of PCI mediated therapy for brain tumors have been initiated. The majority of experimentation to demonstrate the potential of PCI in this area has been limited, with a few exceptions, primarily to in vitro investigation. Nevertheless, the results obtained so far clearly indicate the potential advantages of this technology capable of improving treatment of gliomas.

## 10. Clinical Experience and Future Potential of PCI

### 10.1. Summary of the First-in-Human PCI Trial

**Figure 11:**
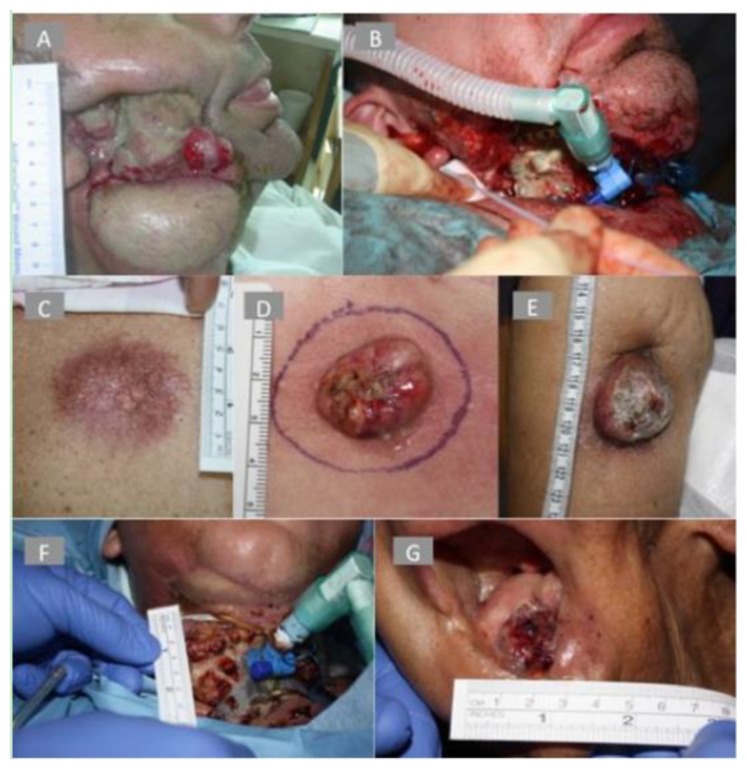
Sample of seven patients that presented with advanced and/or recurrent solid malignancies that were subjected to the PCI surface illumination protocol. (**A**) 56-year-old male with chondroblastic osteosarcoma in the right mandible. The patient has failed multiple surgical resections with reconstructions and multiple rounds of subsequent chemo-radiation. The patient was treated with 0.25 mg/kg TPCS_2a_-induced PCI and had complete response of all the illuminated sarcoma of the R face. (**B**) 45-year-old male with squamous cell carcinoma of the neck with metastasis to the lungs and liver, who failed multiple conventional interventions. The patient was treated with 1.0 mg/kg TPCS_2a_-induced PCI and had partial response (**C**) 61-year-old female with metastatic squamous cell carcinoma (SCC) to the torso (back). The primary malignancy was a tongue base SCC with metastasis to the cervical and axillary lymph nodes. She had multiple failed interventions to the primary site. The patient was treated with 0.5 mg/kg TPCS_2a_-induced PCI and had complete response. (**D**) 46-year-old female with advanced metastatic ductal carcinoma to the torso (anterior) brain, spine, lungs and liver. The patient’s metastatic torso lesions were treated with 0.5 mg/kg TPCS_2a_-induced PCI and had complete response. (**E**) 72-year-old female with metastatic (chemo-resistant) ductal carcinoma to the arm. The primary breast cancer also metastasised to the axillary and cervical lymph nodes. The patient’s metastatic arm lesion was treated with 1.0 mg/kg TPCS_2a_-induced PCI and had partial response. (**F**) 35-year-old male with squamous cell carcinoma of the floor of mouth and neck. This patient failed surgery, chemo-radiation and photodynamic therapy. The cancer metastasised to the right lung and required pneumonectomy. The patient’s extensive disease was treated with 1.0 mg/kg TPCS_2a_-induced PCI and had complete response of the treated areas. (**G**) 73-year-old male with oral squamous cell carcinoma. This patient failed multiple surgical interventions with reconstruction, as well as chemo-radiation. The patient’s extensive disease was treated with 0.5 mg/kg TPCS_2a_-induced PCI and had complete response of the treated areas.

In a recent first-in-human trial conducted at University College London Hospital, 22 patients were recruited with advanced malignancies (recurrent and/or metastatic) (Figure 11) [106]. The study was designed and implemented as an open, phase I dose escalating study to evaluate the safety and tolerance of the disulfonated tetraphenyl chlorin (TPCS_2a_ – the photosensitiser)-induced photochemical internalization with bleomycin as the chemotherapeutic agent. While efficacy was not a primary end point in the study, PCI anti-tumour activity was also documented for future research.

The recruited patients were given TPCS_2a_ on day 0 by slow intravenous injection. The patient was then closely monitored for adverse events and if none was reported, intravenous infusion of Bleomycin (at a fixed dose of 15,000 IU/m) was administered on day 4. An oncologist closely monitored this infusion. After 3 h (on day 4), the surface of a previously selected “target malignant lesion” was illuminated with 652 nm laser light (fixed at 60 J/cm—delivered at an irradiance of 100 mW/cm^2^). A margin was selected beyond the malignant lesion to ensure the elimination of any possible malignant deposits. Furthermore, other macroscopically malignant areas were also subjected to PCI, as appropriate, for the patient’s best interests. The light delivery per treated site lasted approximately 600 s.

The TPCS_2a_ starting dose was 0.25 mg/kg and was then escalated in successive dose cohorts of three patients (0.5, 1.0, and 1.5 mg/kg). The study aimed to try and identify the maximal tolerated dose (MTD). Investigators’ meetings followed the completion of each of the TPCS2a dose cohort, mainly to discuss the data (including adverse events) and to ensure that it was clinically and scientifically safe to escalate the dose further. The patient was excluded from the trial in the event of disease progression or reaching the dose limiting toxicity (DLT) [106].

The resultant data showed that TPCS_2a_ was a safe and tolerable photosensitizer by all patients. The measurement of the vital signs of each patient throughout the trial didn’t reveal any clinical significance. Furthermore, routine blood tests carried out on every patient didn’t reveal any haematological or biochemical profile abnormality. Hence it was safe to conclude that the use of TPCS_2a_ as well as the PCI intervention didn’t have any direct negative effect on any of the monitored organs or processes [106].

**Figure 12:**
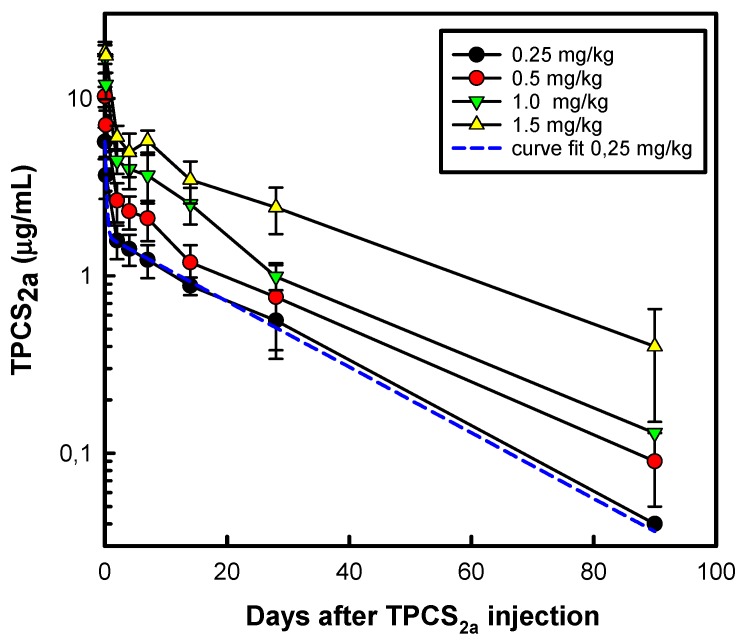
Pharmacokinetic analyses of plasma concentration of TPCS_2a_. The concentration of TPCS_2a_ in plasma samples from patients in the dose cohorts 0.25–1.5 mg/kg was analyzed by fluorescence spectroscopy. The excretion rate of TPCS2a in the 0.25 mg/kg cohort that was regarded as the optimal treatment dose was found to fit well a two-compartment model: *f*(*x*) = 4.43e^−4.71*x*^ + 1.69e^−0.69*x*^ (*R*^2^ = 0.99) as presented in the figure. The figure is modified from previously presented results [106]. Error bars show SD.

The highest mean TPCS_2a_ concentration in the plasma was recorded at the first sample timepoint 30 min after TPCS_2a_ administration for all doses. TPCS_2a_ decreased exponentially fitting well to a two-compartment model (Figure 12). No TPCS_2a_ was detectable in urine [106].

The results illustrated that TPCS_2a_-induced photochemical internalization was an outstanding intervention in such a complex cohort of recurrent advanced malignancies who have exhausted multiple conventional interventions prior to PCI and with a life-expectancy not exceeding few months (Figure 11). Dose-limiting toxicities were reported in two of the treated patients at a TPCS_2a_ dose of 1.5 mg/kg; hence the maximum tolerated dose of TPCS_2a_ was decided to be 1.0 mg/kg. Adverse events related to PCI were classified as local, resulting from the local inflammatory process, or systemic, mostly as a result of the residual photosensitivity. The majority of the local adverse events were related to the advanced malignancy affecting deeper tissues in most patients, which was not treated under the PCI surface illumination therapy protocol. Many of the patients’ symptoms, which were related to the superficial disease subjected to PCI, did improve within a short period of time following the intervention. No treatment-related deaths were recorded [106].

There were unanticipated high levels of intra-illumination and post-illumination pain reported by patients who underwent PCI under local anaesthesia. This was mostly eliminated when the intervention was employed under intravenous sedation or general anaesthesia. This pain was at the site being illuminated and was directly related with the size of the surface area of tumour exposed to illumination and clinical effectiveness [106].

**Figure 13:**
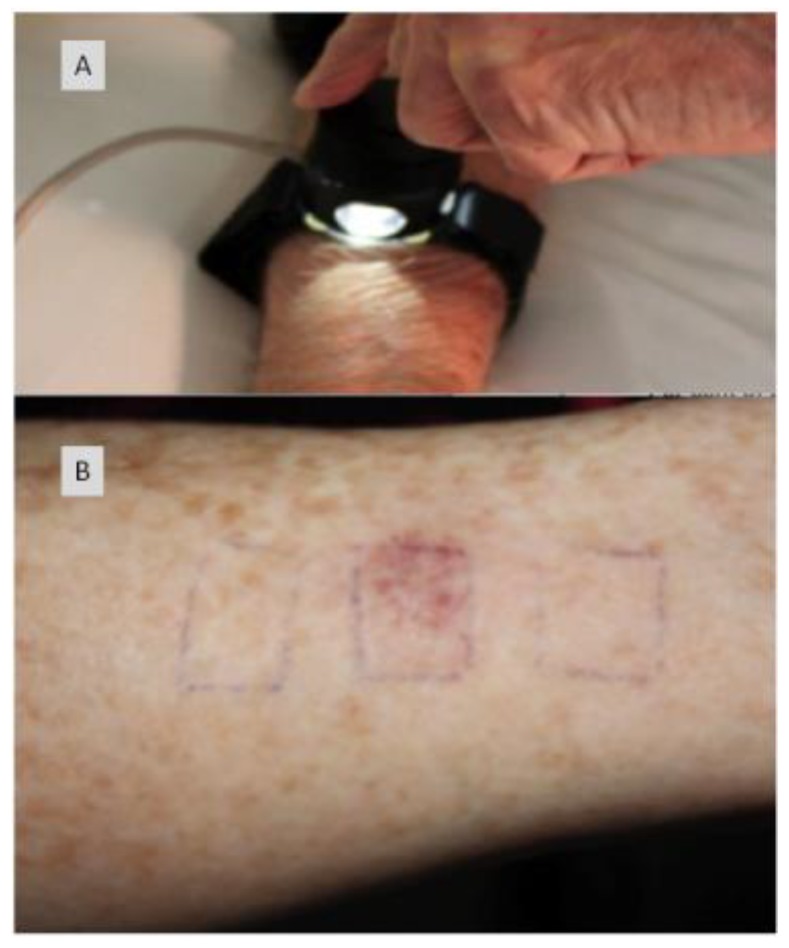
Skin photosensitivity testing in the phase I PCI trial. This was assessed and recorded at definite intervals (days 0, 1, 3, 6, 14, and 28, and at 3 months). Skin photosensitivity tests were undertaken with white light at two intensities: 500 lux and 100,000 lux for durations ranging from 30 s-5 min. Separate 0.8 cm spots on the inside of the arm were exposed to light and patients were assessed at two intervals (1 h and 24 h) after exposure. It was considered vital to report any local skin changes as these could indicate phototoxicity. The scoring of skin photosensitivity was descriptive. (**A**) shows the testing method, (**B**) shows mild skin reaction (erythema). A remarkable discovery from the phase I study was that TPCS_2a_-mediated PCI (of bleomycin) induced convincing tumour responses, even in this heavily pretreated cohort with advanced and/or recurrent malignancies. Although the maximum tolerated dose of TPCS_2a_ was established to be 1.0 mg/kg, robust anti-tumour response was seen at all doses (including the started dose of 0.25 mg/kg). The outcomes suggest that photochemical internalisation had excellent potential for high tumour selectivity when treating cutaneous and subcutaneous malignancies, without causing severe damage to surrounding normal tissue. It was rather unanticipated that complete tumour responses were recorded in cases with malignancies depth of up to 38 mm, which were only treated with surface illumination only [106]. Figure 14 illustrates the PCI process in a sixty-one-year-old female with metastatic squamous cell carcinoma to the torso (back).

One of the main dose-limiting toxic effects was residual skin photosensitivity. Skin sensitivity testing was conducted using white light with a luminance at 500 lux and 50,000 lux (Figure 13). Skin photosensitivity measurements indicated that the reaction appears to be dose dependent (i.e., very mild at 0.25 mg/kg dose and most severe at 1.5 mg/kg dose which was in a patient who did not adhere to post-illumination advice). Skin photosensitivity was reported for a notable period of time following TPCS_2a_ administration, especially at higher doses, but was clinically manageable. The post-illumination advice was gradual sun exposure at an incremental rate of 100 lux per day, and for patients to avoid direct sun exposure for three months after TPCS_2a_ administration. However, exposure to normal daylight (not direct sunlight) is possible after 2–3 weeks [106].

The sample size for each of the dose-related sub-cohorts is considered to be one of the limitations of this trial. Also, a number of patients had to leave the trial before day 28 for a number of reasons, but mainly was to treat other malignant areas (loco-regional or distant) that were not covered under the PCI treatment protocol. Most of the patients had extensive advanced and/or recurrent malignancies, which made it a real challenge to identify a specific “target” lesion and follow-it-up throughout the trial duration. Additionally, the numerous collections of blood and urine samples for pharmacokinetic assessment and the follow-up visits posed some troubles to our cohort with complex medical history. Further, the recruitment of patients for this phase 1 trial was a real challenge with many issues had to be addressed throughout [106].

### 10.2. Case Study

**Figure 15:**
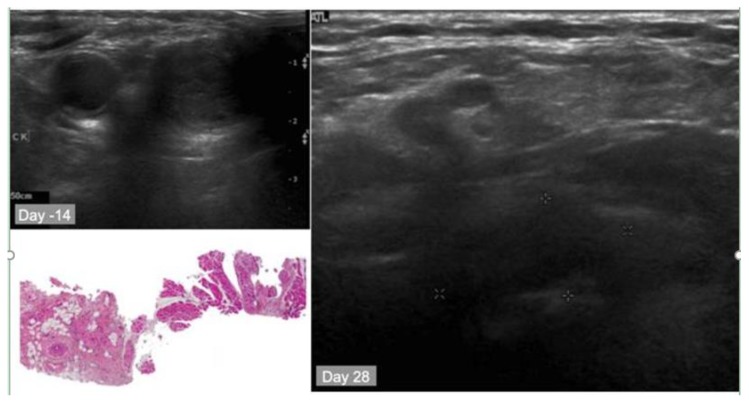
Demonstrates an example of a complete response to bleomycin PCI in a patient with second primary squamous cell cancer of the right glosso-tonsillar sulcus.

A 67-year-old Caucasian male was diagnosed with a second primary squamous cell cancer of the right glosso-tonsillar sulcus (Figure 15). His skin type according to the Fitzpatrick skin classification is III and performance status on the Eastern ECOG Scale was 2. He was initially diagnosed with laryngeal cancer 15 years ago and underwent total laryngectomy and neck dissection of the cervical lymphatic chain and adjuvant radiotherapy. The patient’s medical history includes hypothyroidism, chronic obstructive pulmonary disease and spinal stenosis. He was maintained upon aspirin, simvastatin, Thyroxine, Metformin, Flixotide, Omaprazole, Salbutamol and Aminophylline.

This patient was part of the phase I TPCS_2a_ based Bleomycin PCI trial. Specific data regarding the photosensitiser tolerability and photosensitive reactions were recorded and showed that the patient tolerated the photosensitiser and chemotherapeutic agent well and there were no recorded photosensitive reactions. The patient received 1.1 mL of 0.25 mg/kg TPCS_2a_ by slow intravenous injection (1–6 min) into the median cubital vein, with the patient monitored constantly during this process. Dexamethasone (1 mg intravenously) and chlorphenamine (10 mg intravenously) were given soon afterwards to reduce any potential allergic reaction. The patient was kept in a dimly lit side room to avoid photosensitivity reactions in the skin or the eyes, and monitored closely for adverse events. No clinical changes were expected to tissue at this stage.

Four days later, surface illumination based photochemical internalization (PCI) (652 nm diode laser, 60 J/cm^2^ delivered at irradiance 100 mW/cm^2^) was carried out 3 h after the slow intravenous infusion of Bleomycin (dose of 15,000 U/m^2^ over 15 min). The bleomycin dose was capped at 30,000 U due to patient’s high body surface area (2.513 m^2^). A margin of 10 mm beyond the macroscopic tumour margin was treated to eliminate any infiltration of cancer cells into the tumour microenvironment.

Pre-emptive analgesia was given prior to illumination and no immediate adverse events were reported. Immediate maximum pain scoring post-illumination was 2.6/10 on VAS. Pain was not a problem on the following day (0/10 on VAS). No adverse events were reported by the patient. Complete response of the target lesion was confirmed at 4 weeks post-illumination using ultrasonography, which showed that the carcinoma was non-existent. Surgical core-biopsy under ultrasound guidance was acquired from the treated area and showed no malignancy.

Pre-emptive analgesia was given prior to illumination and no immediate adverse events were reported. Immediate maximum pain scoring post-illumination was 2.6/10 on VAS. Pain was not a problem on the following day (0/10 on VAS). No adverse events were reported by the patient. Complete response of the target lesion was recorded at 4 weeks post-illumination using ultrasonography, which showed that the carcinoma was not detectable. Surgical core-biopsy under ultrasound guidance was acquired from the treated area and showed no malignancy.

Withdrawal from the trial was on Day 36 as the patient required surgical excision of the PCI-treated margins to assess their status. The patient’s performance status on the Eastern ECOG Scale was 2 when he left the trial and remained cancer free for at least 2 years post-PCI.

## 11. Clinical Efficacy of PDT vs. PCI

When it comes to the treatment efficacy in clinical practice, PCI has been more successful in eliminating some of the weaknesses, which we faced with PDT. (A) The treatment margin: in a specific prospective study involving oral SCC, multiple biopsies have been taken from PDT-treated margins in a number of patients four weeks following the intervention indicated the presence of viable tumour deposits which required a second round of treatment. This issue was initially raised in previous clinical studies when a number of patients suffered a treatment margin site recurrence few months post-PDT, despite being given a complete response outcome based on clinical and radiological assessment. When it comes to PCI, the data from the phase I trial clearly indicated that PCI was more effective in treatment margins sites, as all biopsies acquired 4 weeks post-PCI showed necrotic tissue and was tumour-free. (B) The treatment depth: the treatment depth in PDT usually depends on the photosensitizer used and can extend from 2 mm up to 10 mm, with mTHPC. As a result, deep-seated tumours would require multiple treatments and be guided by imaging techniques, which can be time and resource consuming. However, the phase I PCI study showed that treatment depth seems to cross that of the PDT barrier, and in certain cases “depth of effect” reached few centimetres, which is likely related to the chemotherapy release in the deeper tissues. (C) Collateral tissue damage and regeneration: based on clinical observation from treating hundreds of patients with various malignancies, PDT had a problem with collateral tissue damage, and this was present as inflammation, oedema, or even necrosis leaving unnecessary defect which will compromise the regeneration process. Precautions had to be taken to prevent this from happening, but unfortunately it was not possible in every case. On the other hand, PCI appeared to be more tumour-specific with significant reduction in collateral tissue damage, which led to quicker tissue regeneration.

## 12. Summary

PCI has been shown to be a versatile tool for the cytotsolic delivery of endocytosed drugs unable to escape the endolysosomal compartmentalization. The recent and currently ongoing clinical trials are based on PCI activation of approved drugs that are only partly accumulating in lysosomes and therefore not optimal for obtaining high specificity and efficacy. Despite these limitations good clinical outcome has been documented.

TPCS_2a_-based PCI of bleomycin may provide an effective and localised anti-cancer therapy due to the synergistic action from both photodynamic and chemotherapeutic treatments. The first-in-human phase I trial dealt with very difficult-to-treat group of patients who exhausted all the available treatment options of surgery, radiotherapy and chemotherapy. Significant anti-tumour effects were seen with all the doses tested on several different types of tumours in patients with life expectancy not exceeding few months, yet some of them still lived four years after the end of the trial. The PCI-related adverse events were negligible. It was very interesting to see the uniform PCI effect causing tumour death on a number of patients with very aggressive malignancies including squamous cell carcinoma, sarcoma, eccrine (adnexal) carcinoma, and chemo-resistant ductal carcinoma.

The PCI technology, still in the clinical research phase has been used so far in managing advanced and recurrent tumours with high levels of success in eliminating tumours with minimal morbidity and adverse events. Future application of this technology in managing primary disease is yet to come although a phase II pivotal clinical trial on inoperable cholangiocarcinoma has recently been initiated (clinicaltrials.gov identifier NCT04099888).

PCI can be an option specifically when dealing with end-stage disease when the patient has exhausted all conventional treatment options. However, a potential application may also include managing primary disease, which should be considered in future trials. Managing of such an aggressive and widespread form of disease is a real challenge to any clinician. The PCI anti-tumour activity was highly effective and this was proven by randomly surgical biopsies acquired at two different times.

The current experimental and preclinical directions are selecting or designing drugs or drug formuations that are solely activated by PCI. As reviewed here many approaches are under evaluation. The most active area appears to be for PCI to release drugs carried by nanoparticles that in most cases end up in lysosomes. One of the most interesting appears to be the double unwrapping strategy where light of the same wavelength ruptures the endolyosomes and cleaves a photolable bond on the NP simultaneously.

The utilization of immunotoxins may also flourish in combination with PCI. The immunotoxin strategy has struggled with the need to use type II protein toxins that exert an intrinsic ability to penetrate acidic endolysosomal membranes. The specificity will therefore completely rely on the targeting moiety which is usually not fully specific for the cancer cells and thus very few drugs, e.g., mexetumomab, are currently approved for clinical use, but limited by many side effects. The use of immunotoxins based on type I protein toxins will not be dependent on a highly selective targeting moiety and preclinical studies are very promising. PCI of immunotoxins may also be attractive for targeting treatment resistant tumors since these protein toxins are targeting pathways independent of the resistant mechanisms evolving by previous chemo- or other therapies.

PCI has also shown promise in improved antigen presentation at least partly due to better cross-presentation and the results in preclinical and healthy volunteers appear very promising.

The use of PCI to translocate nucleic acids into cytosol and the nucleus is also very promising, depending on the approach in particular in cancer research. It is generally difficult to transfect/transduce all tumor cells so bystander effects in various ways including mRNA expression of tumor antigens in antigen presenting cells may become an interesting approach.

For brain tumors alternative approaches to utilize the PCI technology has been presented as examples on how PCI may be utilized in clinical practice.

The PCI technology has evolved strongly the last 20 years and approximately 70 patient and 90 healthy volunteers have so far been included in evaluating this technology. There is clearly a need for more preclinical and clinical research to develop all the alternative uses of the technology that is encouraged by the promising initial clinical studies.

## 13. Future Directions

The PCI technology, which combines photodynamic therapy and chemotherapy, aims to target multiple pathways to increase response to treatment. This intervention without doubt led to tumour cell death, with minimal collateral damage allowing the healthy tissue to regenerate and restore form and function. The clinical application of this technology in the field of clinical oncology is at its early stages, but the preliminary results are beyond impressive. The uniform effect of PCI against a number of malignancies represents an area of high interest in the molecular genetics of these pathologies. The phase I trial data showed that nearly all the recruited patients had life expectancy not exceeding weeks to few months, yet many have survived for many months to few years after only one round of PCI (average survival about 12 months). Reduction in morbidity and mortality was achieved in many aggressive advanced and/or recurrent malignancies of the head and neck and breast areas. The applications of PCI can extend beyond this to involve other malignancies that are resistant to conventional therapies. Moreover, the role of PCI in managing primary disease is another potential that would allow direct comparisons with chemo-radiation or immunotherapy.

## Figures and Tables

**Figure 14:**
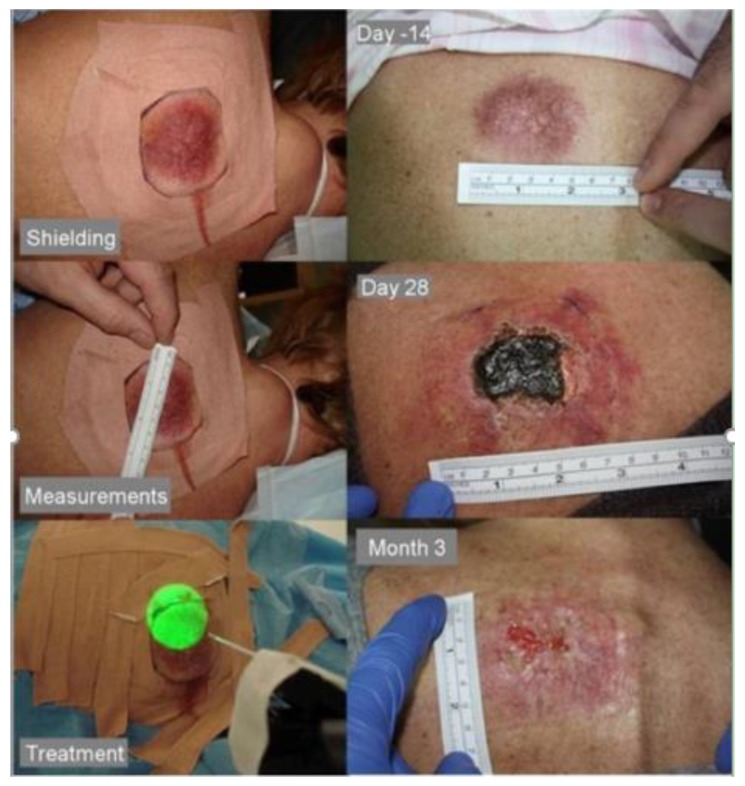
Sixty-one-year-old female with metastatic squamous cell carcinoma (SCC) to the torso (back) treated with PCI of bleomycin. The primary identified disease was in the tongue base with nodal involvement (cervical and axillary). The patient underwent radiotherapy and multiple rounds of chemotherapy for her tongue base carcinoma, which failed to control the disease. Left-hand column: the shielding around the lesion after including 10mm of macroscopically healthy looking tissue to eliminate any risk of micro-infiltration, followed by PCI surface illumination. Right hand column: the “target lesion” on day -14, day 28 and month 3, where complete response was achieved. For month 3, selective surgical biopsies acquired from the centre and peripheries were found to be tumour-free.
